# Uncertainty Quantification for Free Energy Calculations by Generalized Hierarchical Bayesian Inference

**DOI:** 10.1021/acs.jctc.6c01533

**Published:** 2026-10-21

**Authors:** Martin Skorna, Adam Gottfried, Zuzana Janackova, Katarina Baxova, Pavel Jungwirth, Brennon L. Shanks

**Affiliations:** Institute of Organic Chemistry and Biochemistry of the Czech Academy of Sciences, Flemingovo nám. 2, 166 10 Prague 6, Czech Republic

## Abstract

Free energy calculations are routinely used to study molecular processes inaccessible to unbiased molecular dynamics, but their utility ultimately depends on knowing when and how much their predictions can be trusted. Uncertainty estimation is therefore essential for distinguishing genuine physical features of a free energy profile from artifacts arising from limited simulation data or inadequate sampling. Gaussian processes have emerged as a powerful framework for reconstructing free energy profiles together with predictive uncertainties. However, existing implementations typically condition on fixed hyperparameters and observation noise, preventing predictive uncertainties from adapting to the information content of the simulation data. Here, we develop a generalized hierarchical Gaussian process framework that accounts for these neglected sources of uncertainty. Applications to umbrella sampling and extended Lagrangian metadynamics of peptide-lipid membrane interactions demonstrate that the resulting uncertainty estimates track reconstruction errors across a wide range of sampling and data conditions.

## Graphical abstract

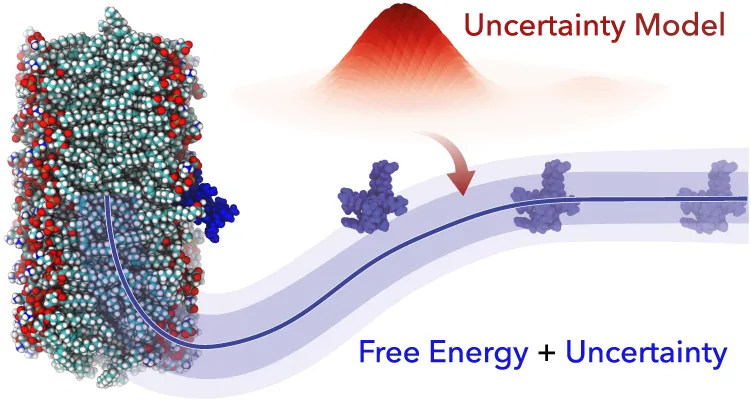

## Introduction

Enhanced-sampling free energy calculations are among the most widely used applications of molecular dynamics (MD), providing a powerful framework for understanding atomic-scale interactions and thermodynamic processes in molecular systems. By projecting the high-dimensional configurational phase space of a molecular system onto a reduced set of coordinates, free energy calculations facilitate the study of molecular processes whose characteristic time scales lie beyond the reach of unbiased MD. Over the past several decades, the development of enhanced sampling techniques such as umbrella sampling,^1–3^ metadynamics,^4,5^ and related methods^6–9^ has dramatically expanded the range of systems that can be studied computationally.

A fundamental challenge of free energy methods is principled uncertainty quantification (UQ), which aims to estimate both the free energy profile and its uncertainty. This problem is especially challenging for short simulation trajectories or sparse sampling. In such low-data regimes, rigorous UQ would enable adaptive sampling strategies that avoid oversampling of well-characterized regions and under-sampling near barriers or transition states, thus saving valuable computational effort and enabling automated computational workflows. However, standard UQ approaches rely on assumptions that become questionable in precisely these regimes. For example, error propagation for variance estimators with sparse umbrella window centers, given a normal distribution approximation for the biased distributions, underestimates the total sampled error.^10^ On the other hand, block averaging and bootstrapping methods require fully equilibrated trajectories and user-specified choice of the number of independent trajectory epochs, which significantly impacts the estimated predictive variance.^11^ While these approaches can be effective under ideal sampling conditions, their uncertainty estimates are less informative when data are limited, precisely where accurate reliable UQ is most important.

To address these limitations, we employ a Gaussian process (GP), which treats free energy reconstruction as a problem of statistical inference. In a nutshell, the method assumes the free energy is an unknown function of the collective variables, and begins with a broad range of physically plausible functions that satisfy basic assumptions such as continuity and differentiability. This initial description, known as the *prior*, represents our knowledge before obtaining simulation data. MD simulations then provide incomplete information about this unknown free energy, and Bayesian inference quantifies the probability of different functions as new data is incorporated. The final result is a probability distribution, known as the *posterior*, that identifies the most plausible free energy functions given both the prior assumptions and the simulation data. GPs have been used for umbrella sampling,^12^ metadynamics,^13^ mulistate Bennet acceptance ratio,^14^ and the interpolation of quantum potential energy surfaces.^15,16^ Bayesian optimization for umbrella sampling, which leverages a GP as a guide for biasing window selection, has even been shown to improve sampling efficiency by a factor of 1.6–2.8 across a diverse set of two-dimensional systems.^17^

While existing GP-based free energy estimators have been successfully applied across multiple areas of computational chemistry, rigorous evaluation of their UQ performance remains limited. A key unresolved issue is the treatment of hyperparameters, which are GP model parameters that specify properties of the prior. Earlier work has either fixed hyperparameters *a priori* based on empirical evidence of low root-mean-square-error (RMSE) between the GP mean and “ground truth”, or varied hyperparameters to assess sensitivity *post hoc*. As we will show, assuming GP hyperparameters neglects important sources of uncertainty and can prevent the model from adapting as new simulation data are acquired. Consequently, the resulting uncertainty estimates are often misleading and do not accurately reflect the true level of uncertainty in the reconstruction.

Here, we develop a generalized hierarchical GP framework for free energy reconstruction that provides reliable uncertainty estimates that respond consistently to data quality and availability. The method combines a joint function-derivative GP observation model with Hamiltonian Monte Carlo No–U-Turn Sampling (HMC-NUTS) of a generalized hyperposterior to propagate uncertainty in the GP hyperparameters to the free energy profile. Using the free energy profile of interaction of a nonaarginine (R9) peptide with a lipid bilayer as a benchmark, an important system for understanding the membrane penetration mechanism of cell-penetrating peptides (CPPs), we find that several commonly employed UQ strategies can produce misleading assessments of convergence, either by exhibiting little sensitivity to additional data or by becoming overconfident as sampling progresses. In contrast, the hierarchical Bayesian approach avoids these pathological behaviors and yields uncertainties that systematically evolve with increasing data acquisition. Together, these results highlight the importance of hyperparameter uncertainty in free energy UQ and suggest a practical path toward uncertainty-aware and automated free energy workflows.

## Methods

Enhanced sampling simulations provide samples of the biased probability distribution of a molecular system along a collective variable (CV), **ξ**, which serve as input data **y** for GP reconstruction of the free energy profile (Figure 1).

**Figure 1:**
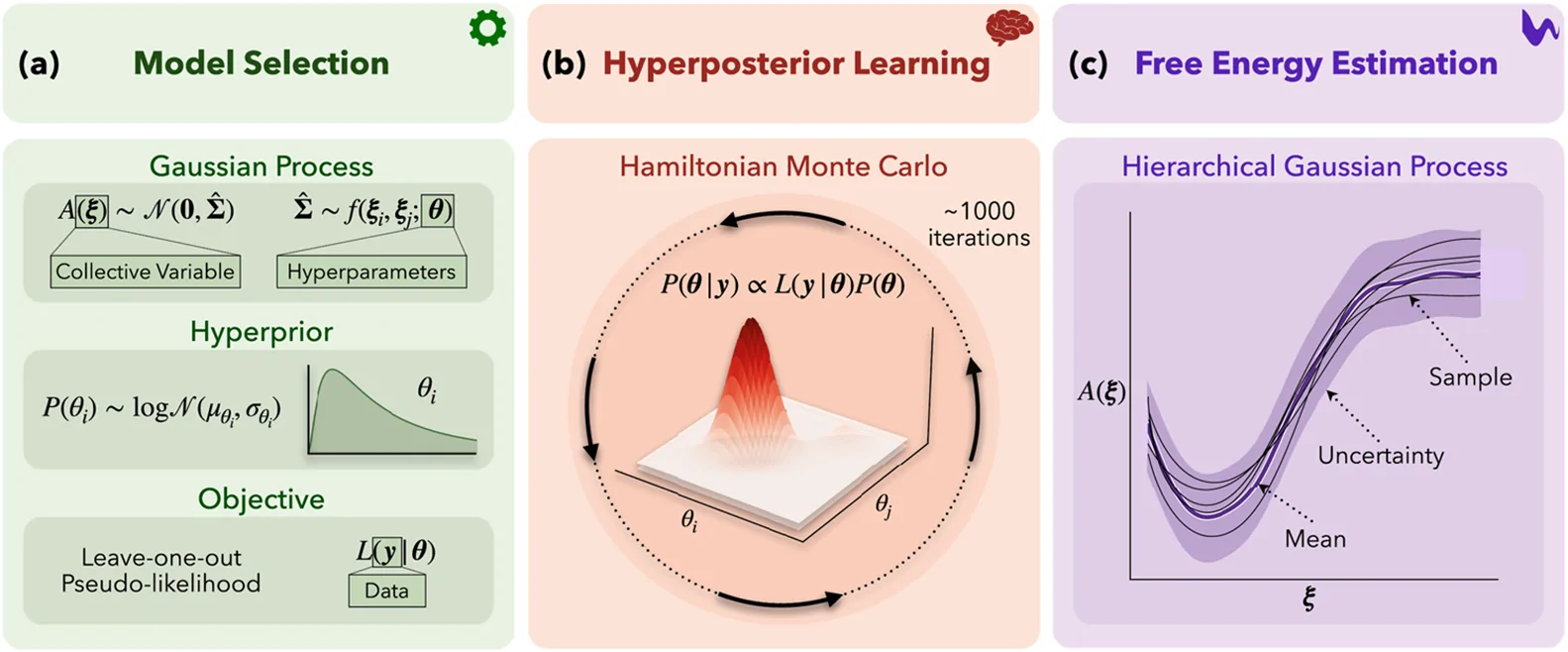
Flowchart summarizing the workflow of the generalized hierarchical Bayesian framework for free energy reconstruction. (a) Model selection includes choosing the GP prior mean and kernel, hyperpriors, and likelihood objective. (b) Hamiltonian Monte Carlo is used to sample the resulting GP hyperposterior. (c) Hyperposterior uncertainty is propagated to the free energy profile by sampling the hyperposterior, evaluating the conditional GP predictive distribution for each sample, and approximating the resulting mixture of Gaussians by its first two moments (mean and variance).

Model selection (Figure 1a) is the process of specifying a probabilistic model for free energy inference. This involves choosing the GP prior mean and covariance functions, which depend on unknown parameters called *hyperparameters*. Priors on these quantities, called *hyperpriors*, constrain nonphysical behavior while allowing the model sufficient flexibility to learn from the MD data. Because priors are specified for both the free energy profile and the GP hyperparameters, the method is *hierarchical*, consisting of multiple levels of uncertainty that are ultimately propagated to the final prediction. The final component of model selection is the choice of *likelihood*, which quantifies how well a model explains the observed data. Here, we employ a leave-one-out (LOO) pseudolikelihood that favors models with strong predictive performance on held-out observations.

Hyperposterior learning (Figure 1b) is the process of sampling the unknown probability distribution given the model and data. The unnormalized *hyperposterior*, the posterior distribution over the GP hyperparameters, is given by Bayes’ theorem as the product of the hyperpriors and the likelihood. With the LOO pseudolikelihood, this object is technically not a proper hyperposterior, but rather a pseudo- or generalized hyperposterior. The resulting distribution is not available in closed form and is therefore sampled via HMC-NUTS.

Free energy reconstruction (Figure 1c) is performed by propagating samples from the generalized hyperposterior through the GP predictive equations, yielding predictive credible intervals for the free energy surface. The result is commonly summarized by the posterior mean and a ±σ or ±2σ credible interval, although posterior samples can also be propagated to quantify uncertainty in downstream observables such as free energy barriers. The resulting free energy posterior therefore reflects uncertainty arising from both the molecular simulation data and the inferred GP hyperparameters.

### Free Energy Estimation

The first stage of the workflow is the construction of observations of the free energy profile from enhanced sampling MD simulations. Because the underlying theory and practical implementation of free energy methods are well documented elsewhere,^18^ we restrict ourselves to a brief discussion of the approaches used in the present work: umbrella sampling and extended Lagrangian metadynamics.

#### Umbrella Sampling

In umbrella sampling, a system is simulated under a set of biased potentials centered at prescribed locations **ξ**_*w*_ in CV space. A CV is a function of the microscopic coordinates chosen to describe the molecular process of interest, such as the distance or angle between two species. One or more CVs define the reduced coordinate space used for free energy reconstruction. For a harmonic umbrella window *w* with force constant κ_*w*_, the biasing potential is

1 $$\psi^{w}\left(\xi \right)=\frac{1}{2}\kappa_{w}{\left(\xi -\xi_{w}\right)}^{2}$$

The biased simulations generated using equation 1 provide two complementary sources of information: biased probability histograms and local mean-force estimates.

Umbrella sampling provides empirical observations of the free energy function values, up to an additive constant. For bin *i* centered at **ξ**_*i*_, the unbiased free energy is related to the biased histograms by

2 $$A\left(\xi_{i}\right)=-\beta^{-1}\log P^{w}\left(\xi_{i}\right)-\psi^{w}\left(\xi_{i}\right)+c_{w}$$

where *P* ^*w*^(**ξ**_*i*_) is a histogram-based estimate of the biased probability density at bin *i* from window *w* and *c*_*w*_ is an unknown window-dependent additive constant arising from the normalization of the biased distribution. In the weighted histogram analysis method (WHAM), these constants are determined self-consistently across all windows to reconstruct a global unbiased probability distribution.^2^ In the present framework, we instead treat the window-dependent constants in equation 2 as nuisance GP hyperparameters and marginalize them out analytically, while using the histogram-derived values as local observations of the free energy profile.

The free energy gradient at window *w* can be estimated from the mean restraining force in the biased simulation

3 $${\frac{\partial A\left(\xi \right)}{\partial \xi }|}_{\xi_{w}}\approx -{\left\langle \kappa_{w}\left(\xi -\xi_{w}\right)\right\rangle }_{w}$$

where ⟨·⟩_*w*_ is the mean of the biased distribution sampled in window *w*. The quantities obtained from equation 3 are treated as free energy derivative observations, in a manner analogous to the umbrella integration method.^3^

#### Metadynamics

Metadynamics is an enhanced sampling method in which the system is biased along a set of CVs by depositing history-dependent Gaussian potentials centered on previously visited CV values. As the simulation progresses, the accumulated bias progressively destabilizes previously explored free energy minima, encouraging transitions into neighboring regions of CV space. Under appropriate conditions, the accumulated bias converges to the negative of the underlying free energy profile (up to a constant).

In extended Lagrangian formulations, auxiliary variables ***z*** are introduced and coupled to the CVs through a stiff harmonic restraint^5^

4 $$V_{\mathrm{coupling}}\left(\xi ,z\right)=\frac{1}{2}\kappa {\left(\xi -z\right)}^{2}$$

while the history-dependent bias is applied only in the auxiliary space. Under sufficiently stiff coupling in equation 4, ***z*** ≈ **ξ**, and

$${\frac{\partial A}{\partial z}|}_{z*}={\left\langle \frac{\partial V_{\mathrm{coupling}}}{\partial z}\right\rangle }_{z*}=-{\left\langle \kappa \left(\xi -z\right)\right\rangle }_{z*}$$ 5

The physical potential does not depend explicitly on ***z***, hence the estimator reduces to the derivative of the coupling term alone. Equation 5 therefore provides direct estimates of the mean force at sampled values of ***z***, which are treated as noisy derivative observations of the free energy profile.

Histogram observations are obtained by a discrete binning of neighboring ***z*** to construct an umbrella biased histogram of the corresponding **ξ** values. The binning procedure, which includes choosing the number of bins and the bin width, adds an additional layer of uncertainty.

### Gaussian Process Regression

The core of our approach is to model the free energy profile *f*(**ξ**) over the CV space $\xi \in R^{n}$ as a zero mean GP

6 $$f∼\mathrm{GP}\left(0,k\left(\xi ,\xi^{\prime }\right)\right)$$

so that for a set of test inputs *X* ^_*_ = [**ξ**_1_, ..., **ξ**_N_*__], the latent function values f_*_ = [*f*(**ξ**_*i*_)]_*i* = 1_^N_*_^ follow a zero mean multivariate normal distribution with covariance matrix ***K* ^**(*X* ^_*_, *X* ^_*_), where [***K* ^**]_*ij*_ = *k*(**ξ**_*i*_,**ξ**_*j*_) are elements governed by the kernel function. Equation 6 therefore defines the GP prior over the latent free energy function *f*.

Given noisy free energy observations ***y*** = [*y*_1_,...,*y*_*N*_] at inputs ***X* ^** = [**ξ**_1_,...,**ξ**_*N*_], the standard GP posterior mean and covariance is

7 $${\mathrm{f̅}}_{*}=\mathrm{K̂}\left({\mathrm{X̂}}_{*},\mathrm{X̂}\right){\left[\mathrm{K̂}\left(\mathrm{X̂},\mathrm{X̂}\right)+\sigma_{n}^{2}Î\right]}^{-1}y$$

8 $$\begin{array}{l} \mathrm{cov}\left[f_{*}\right]=\mathrm{K̂}\left({\mathrm{X̂}}_{*},{\mathrm{X̂}}_{*}\right)-\mathrm{K̂}\left({\mathrm{X̂}}_{*},\mathrm{X̂}\right)\cdot {\left[\mathrm{K̂}\left(\mathrm{X̂},\mathrm{X̂}\right)+\sigma_{n}^{2}Î\right]}^{-1}\mathrm{K̂}\left(\mathrm{X̂},{\mathrm{X̂}}_{*}\right) \end{array}$$

#### Inference from Free Energy Histograms

To infer the free energy profile from biased histograms in a manner analogous to WHAM, unknown additive constants associated with individual umbrella windows must be accounted for. Following established GP formulations, we represent these offsets through an explicit linear basis in the GP mean. For each bin center **ξ**_*i*_, we define a characteristic basis vector

9 $$h\left(\xi_{i}\right)=\left[\delta_{\xi_{i}}\left(w\right)|w=1,...,N_{w}\right]$$

where

10 $$\delta_{\xi_{i}}\left(w\right)=\{\begin{array}{ll} 1, & \xi_{i}\in w\\ 0, & \xi_{i}\notin w \end{array}$$

indicates whether bin *i* belongs to window *w*. The corresponding weights **β** are the unknown window-dependent offsets. Collecting the basis vectors defined by equations 9 and 10 for all training and test points gives the design matrices ***H* ^** and *H* ^_*_, respectively, with entries [***H* ^**]_*iw*_ = 1 if bin *i* belongs to window *w* and zero otherwise.

The observation model for histogram-derived free energy values is written as

11 $$y=Ĥ\beta +f\left(\mathrm{X̂}\right)+\varepsilon ,,\varepsilon ∼N\left(0,{\mathrm{\Sigma ̂}}_{y}\right)$$

where **β** represents the unknown window-dependent offsets. These “nuisance” parameters are assigned a Gaussian prior and analytically marginalized, yielding a modified GP posterior. As shown by O’Hagan,^19^ in the limit of an uninformative prior over **β**, the posterior predictive distribution resulting from the observation model in equation 11 remains Gaussian, with mean and covariance

12 $${\mathrm{g̅}}_{*}={\mathrm{f̅}}_{*}+{\mathrm{R̂}}^{T}\mathrm{\beta ̅},,\mathrm{cov}\left[g_{*}\right]=\mathrm{cov}\left[f_{*}\right]+{\mathrm{R̂}}^{T}{Ŝ}^{-1}\mathrm{R̂}$$

where

13 $$\begin{array}{l} \mathrm{R̂}={Ĥ}_{*}^{T}-{Ĥ}^{T}{\left[\mathrm{K̂}\left(\mathrm{X̂},\mathrm{X̂}\right)+\sigma_{f}^{2}Î\right]}^{-1}\mathrm{K̂}\left(\mathrm{X̂},{\mathrm{X̂}}_{*}\right)\\ \mathrm{\beta ̅}={\left({Ĥ}^{T}{\left[\mathrm{K̂}\left(\mathrm{X̂},\mathrm{X̂}\right)+\sigma_{f}^{2}Î\right]}^{-1}Ĥ\right)}^{-1}{Ĥ}^{T}{\left[\mathrm{K̂}\left(\mathrm{X̂},\mathrm{X̂}\right)+\sigma_{f}^{2}Î\right]}^{-1}y\\ Ŝ={Ĥ}^{T}{\left[\mathrm{K̂}\left(\mathrm{X̂},\mathrm{X̂}\right)+\sigma_{f}^{2}Î\right]}^{-1}Ĥ \end{array}$$

The additional terms in equations 12 and 13 relative to equations 7 and 8 account for the uncertainty associated with the unknown window-dependent offsets.

#### Inference from Free Energy Gradients

The same framework can be extended to noisy derivative observations, the main difference being that we no longer need to consider the unknown additive constants arising from the biased probability distributions (they become zero after differentiation). Let ***y*** ′ = [*y*_1_^′^,...,*y*′_*M*_] denote gradient observations associated with input locations ***X* ^**′ = [**ξ**_1_^′^,...,**ξ**_*M*_^′^]. Since differentiation is linear, derivatives of a GP remain jointly Gaussian. Defining the structured derivative operators

14 $${\left[\partial_{i}\mathrm{K̂}\left(\mathrm{X̂},\mathrm{X̂}\right)\right]}_{\mathrm{ij}}=\nabla_{\xi_{i}}k\left(\xi_{i},\xi_{j}\right)$$

and

15 $${\left[\partial_{i}\partial_{j}\mathrm{K̂}\left(\mathrm{X̂},\mathrm{X̂}\right)\right]}_{\mathrm{ij}}=\nabla_{\xi_{i}}\nabla_{\xi_{j}}k\left(\xi_{i},\xi_{j}\right)$$

the posterior predictive distribution for derivative-informed inference written in terms of equations 14 and 15 is Gaussian with mean and covariance

16 $${\mathrm{f̅}}_{*}=\partial_{j}\mathrm{K̂}\left({\mathrm{X̂}}_{*},{\mathrm{X̂}}^{\prime }\right){\left[\partial_{i}\partial_{j}\mathrm{K̂}\left({\mathrm{X̂}}^{\prime },{\mathrm{X̂}}^{\prime }\right)+\sigma_{d}^{2}Î\right]}^{-1}y^{\prime }$$

17 $$\mathrm{cov}\left[f_{*}\right]=\mathrm{K̂}\left({\mathrm{X̂}}_{*},{\mathrm{X̂}}_{*}\right)-\partial_{j}\mathrm{K̂}\left({\mathrm{X̂}}_{*},{\mathrm{X̂}}^{\prime }\right)\cdot {\left[\partial_{i}\partial_{j}\mathrm{K̂}\left({\mathrm{X̂}}^{\prime },{\mathrm{X̂}}^{\prime }\right)+\sigma_{d}^{2}Î\right]}^{-1}\partial_{j}\mathrm{K̂}{\left({\mathrm{X̂}}_{*},\mathrm{X̂}\right)}^{\prime T}$$

Equations 16 and 17 therefore give the GP predictive mean and covariance conditioned on the free energy gradients.

#### Joint Histogram-Gradient Inference

Both observation types can be incorporated into a single GP model by concatenating the observation vectors, ***y*** ⊕ ***y*** ′, and constructing the joint latent covariance matrix

18 $${\mathrm{K̂}}_{y⊕y\prime }=\left(\begin{array}{ll} \mathrm{K̂}\left(\mathrm{X̂},\mathrm{X̂}\right) & \partial_{j}\mathrm{K̂}\left(\mathrm{X̂},{\mathrm{X̂}}^{\prime }\right)\\ \partial_{i}\mathrm{K̂}\left({\mathrm{X̂}}^{\prime },\mathrm{X̂}\right) & \partial_{i}\partial_{j}\mathrm{K̂}\left({\mathrm{X̂}}^{\prime },{\mathrm{X̂}}^{\prime }\right) \end{array}\right)$$

The corresponding test-training cross-covariance is

19 $${\mathrm{K̂}}_{*,y⊕y\prime }=\left[\mathrm{K̂}\left({\mathrm{X̂}}_{*},\mathrm{X̂}\right)\partial_{j}\mathrm{K̂}\left({\mathrm{X̂}}_{*},{\mathrm{X̂}}^{\prime }\right)\right]$$

The joint observation noise covariance is modeled as

20 $${\mathrm{\Sigma ̂}}_{y⊕y\prime }=\left(\begin{array}{ll} {\mathrm{\Sigma ̂}}_{y} & Ô\\ Ô & {\mathrm{\Sigma ̂}}_{y\prime } \end{array}\right)$$

where **Σ̂**_***y***_ and **Σ̂**_***y*** ′_ denote the covariance matrices associated with the histogram and derivative observations, respectively. In equation 20, the histogram and derivative observation noise are assumed to be uncorrelated, so the off-diagonal blocks are zero.

The corresponding joint observation covariance is therefore

21 $${Ĉ}_{y⊕y\prime }={\mathrm{K̂}}_{y⊕y\prime }+{\mathrm{\Sigma ̂}}_{y⊕y\prime }$$

equation 21 thus combines the latent covariance of equation 18 with the observation noise covariance of equation 20.

Although the latent free energy and its derivative are correlated through the GP cross-covariance blocks, correlations may also arise between observation errors because the histogram and derivative estimates are obtained from the same simulation trajectories. These correlations are neglected in the present model. If substantial, this approximation could lead to overconfident uncertainty estimates. Explicitly estimating these cross-covariances from the simulation trajectories would provide a more complete noise model and represents a possible extension of the present framework.

The unknown window-dependent offsets apply only to the histogram observations and vanish for derivative observations. The posterior predictive mean and covariance then follow directly from equations 7 and 8 by replacing the observation vector ***y*** with ***y*** ⊕ ***y*** ′, the training covariance ***K* ^**(***X* ^**, ***X* ^**) + **Σ̂**_***y***_ with ***C* ^**_***y*** ⊕ ***y*** ′_, and the test-training cross-covariance ***K* ^**(*X* ^_*_, ***X* ^**) with *K* ^_*, ***y*** ⊕ ***y*** ′_ defined in equation 19.

### Specifying the Gaussian Process Prior

At this stage, the posterior GP is fully specified up to the choice of covariance kernel. The kernel function encodes the prior assumptions that govern the smoothness, correlation structure, and uncertainty of the free energy profile, and are selected based on known physical behaviors of the underlying latent function. Free energy profiles are generally assumed to be continuous and at least twice differentiable (*C* ^2^) in physically meaningful collective variables (such as distances, angles, coordination numbers, etc.), which is sufficient for defining mean forces (first derivatives) and local curvature (second derivatives).

Here we model the free energy as a smooth, or *C* ^∞^, function that is continuously differentiable up to any order derivative with the squared-exponential (or radial basis function) kernel

22 $$k_{\mathrm{SE}}\left(\xi ,\xi^{\prime }\right)=w^{2}\exp\left(-\frac{{\left|\xi -\xi^{\prime }\right|}^{2}}{2l^{2}}\right)$$

where l is a length scale and *w* is the kernel amplitude. Equation 22 enforces a stronger regularity than is strictly required for defining mean forces or local curvature. Other kernels for GP regression tasks are well described in Rasmussen and Williams^20^ and were not investigated in this work.

#### A Note on Noise

Noise is typically estimated directly from molecular simulation statistics, for example using histogram-based variance estimators. Implicit in this approach is the assumption that the GP prior is sufficiently expressive to represent the free energy profile, such that any residual discrepancy between the observations and the GP model arises solely from statistical uncertainty in the simulation. In practice, however, residuals may also reflect model mismatch arising from limitations of the chosen GP prior. Consequently, fixing the noise from simulation statistics alone attributes all model-data discrepancy to sampling uncertainty, potentially leading to pathological hyperparameter inference and misleading uncertainty estimates.

We address this limitation by treating the noise term as an unknown hyperparameter and inferring it directly from the data. Under this interpretation, the noise represents an effective measure of data-model mismatch rather than pure measurement error, incorporating simulation uncertainty, temporal and spatial correlations not represented explicitly in the diagonal noise approximation, numerical errors, and fluctuations in the data that the GP regression may not be fully equipped to explain. As we will show, this simple reinterpretation prevents the optimizer from converging to nonphysical hyperparameter configurations and yields uncertainty estimates that respond appropriately to data acquisition.

### Generalized Hyperposterior Learning

Previous work suggested that free energy profile reconstruction is relatively insensitive to hyperparameter selection, leading to the use of fixed hyperparameters chosen *a priori*. In practice this approach can be well-justified for estimating the profile mean, particularly if one already has strong prior knowledge of the behavior of the underlying function. However, selecting hyperparameters based on expert knowledge effectively fixes predictive uncertainty, preventing adaptive response of the UQ to new data.

We therefore relax the assumption that the GP hyperparameters can be specified *a priori*. A common alternative is to optimize them from observed data by maximizing the GP marginal likelihood (or, more generally, the posterior over hyperparameters), yielding a *maximum a posteriori* (MAP) estimate. Although MAP estimation is computationally efficient compared to sampling the full hyperposterior with Monte Carlo methods, it conditions the predictive distribution on a single set of hyperparameters and therefore neglects their uncertainty. This approximation can lead to underdispersed predictive uncertainty, particularly when the available data are limited.^20^

Rather than conditioning the GP on a single optimized set of hyperparameters, we construct and sample the full hyperparameter distribution, yielding a hierarchical Bayesian model. The target distribution is defined by a physically motivated hyperprior combined with a leave-one-out (LOO) cross-validation pseudolikelihood,^21^ which has previously been shown to improve uncertainty quantification in computational chemistry applications.^22,23^ In the present work, the LOO objective also produced more informative uncertainty estimates than the standard GP marginal likelihood.

Because the LOO objective is not a proper likelihood, the resulting distributions are formally pseudoposteriors (or generalized posteriors) rather than exact Bayesian hyperposteriors. For simplicity, we retain the term “hyperposterior” throughout the remainder of the manuscript. Complete specifications of the GP mean functions, covariance kernels, and hyperpriors used in each analysis are provided in Supporting Information Section S1.

#### Hamiltonian Monte Carlo Implementation

Hamiltonian Monte Carlo with the No–U-Turn Sampler (HMC-NUTS), as implemented in Pyro,^24^ was used for hyperposterior sampling. The target density was the sum of the log hyperprior and the LOO pseudolikelihood of the joint histogram-derivative GP model. Positive kernel and noise hyperparameters were sampled in unconstrained log space and transformed back by exponentiation within the probabilistic model, the so-called “log trick”. For each proposed hyperparameter state, the full joint covariance matrix of the function and derivative observations was assembled in PyTorch, regularized with a small diagonal “jitter” term for numerical stability (10^–8^), and factorized via Cholesky decomposition to evaluate the target log density. Gradients required for leapfrog integration were obtained by automatic differentiation through the GP matrix operations.

An initial burn-in phase of 500 steps was used to adapt the HMC step size and mass matrix, followed by a production phase of 1000 retained samples, which was sufficient for stable estimation of free energy observables with four independent NUTS chains (see Supporting Information Section S2). Chains were initialized from dispersed random starting points and run in parallel. Convergence was assessed using standard multichain Markov chain Monte Carlo diagnostics extracted from the Pyro sampler output, including the split potential scale reduction factor (*R̂*), effective sample size (ESS), divergence counts, and chain-resolved trace plots and marginal posterior histograms for each sampled hyperparameter. We defined convergence when *R̂* < 1.01 and bulk and tail ESS exceeded 200 for all parameters (with no persistent divergences). We note that standard HMC convergence diagnostics do not necessarily ensure complete exploration of the hyperposterior and may fail to detect unvisited modes in multimodal parameter spaces, but this effect is mitigated by initiating chains in different regions of the hyperparameter space.

#### Hyperposterior Uncertainty Propagation

The propagation of hyperparameter uncertainty can be understood through the GP predictive distribution at test inputs *X* ^_*_ conditioned on the hyperparameters

23 $$p\left(f_{*}|y,\theta \right)=N\left(\mu_{\theta },{\mathrm{\Sigma ̂}}_{\theta }\right)$$

where $\mu_{\theta }\in R^{N_{*}}$ and ${\mathrm{\Sigma ̂}}_{\theta }\in R^{N_{*}\times N_{*}}$ are the GP posterior mean and covariance. To obtain the hyperparameter-marginalized predictive distribution, we marginalize the conditional GP predictive distribution over the generalized hyperposterior, *p*(**θ** | ***y***)­

24 $$p\left(f_{*}|y\right)=\int p\left(f_{*}|y,\theta \right)p\left(\theta |y\right)d\theta$$

which yields a mixture of Gaussian distributions. Although this hierarchical predictive distribution is not Gaussian in general, its first and second moments are given in closed form by the laws of total expectation and total variance^25^

25 $$\mathrm{\mu ̅}=E\left[f_{*}|y\right]=E_{\theta |y}\left[\mu_{\theta }\right]$$

26 Σ̅=Cov(f*|y)=Eθ|y[Σ̂θ]+Covθ|y(μθ)

or equivalently,

27 $$\mathrm{\Sigma ̅}=E_{\theta |y}\left[{\mathrm{\Sigma ̂}}_{\theta }\right]+E_{\theta |y}\left[\mu_{\theta }\mu_{\theta }^{Τ}\right]-\mathrm{\mu ̅}{\mathrm{\mu ̅}}^{Τ}$$

Given *M* samples {**θ** ^(*m*)^}_*m* = 1_^*M*^ from *p*(**θ** | ***y***), we approximate equations 25–27 via

28 $$\mathrm{\mu ̅}\approx \frac{1}{M}\sum_{m=1}^{M}\mu_{\theta^{\left(m\right)}}$$

29 $$\mathrm{\Sigma ̅}\approx \frac{1}{M}\sum_{m=1}^{M}{\mathrm{\Sigma ̂}}_{\theta^{\left(m\right)}}+\frac{1}{M}\sum_{m=1}^{M}\left(\mu_{\theta^{\left(m\right)}}-\mathrm{\mu ̅}\right){\left(\mu_{\theta^{\left(m\right)}}-\mathrm{\mu ̅}\right)}^{Τ}$$

The average posterior mean of equation 28 is just the average of the sampled means. The first term in equation 29 represents the average posterior variance conditioned on **θ**, while the second term accounts for the uncertainty in the GP hyperparameters. This hyperposterior uncertainty propagation step therefore propagates uncertainty arising from both the GP prediction and the inferred hyperparameters into the reconstructed free energy profile.

Note, however, that the interpretation of this uncertainty is subtle because only free energy differences are physically observable, leaving the free energy profile defined only up to an arbitrary additive constant. The resulting predictive covariance therefore reflects uncertainty only in the relative free energy profile, independent of the arbitrary choice of energy zero. In practical terms, this uncertainty characterizes the physically meaningful features of the landscape, including the relative depths of metastable states and the free energy barriers separating them.

### Uncertainty under Data Ablation

To assess how uncertainty changes as a function of the amount of available data, we performed free energy UQ while systematically knocking-out umbrella windows and trajectory time. As a test system, we studied umbrella sampling data of a nonaarginine peptide­(R9) interacting with a lipid bilayer. The CV was defined as the distance between the centers of mass of the membrane and peptide normal to the membrane surface. The data set consists of 25 umbrella windows, each constrained by a harmonic potential with ∼200 ns long trajectories. Convergence of the reference free energy profile with WHAM is assessed in the Supporting Information Section S3 and simulation details are described elsewhere.^26^

The four methods presented are (a) umbrella integration (UI) with block averaging (methodology in Supporting Information Section S4), (b) a fixed hyperparameter GP regression model with simulation derived noise variances, (c) our hierarchical GP model with optimized hyperparameters (MAP estimation), and (d) the same hierarchical GP but with hyperposterior uncertainty propagation. We also evaluated BayesWHAM, a Bayesian formulation of WHAM that infers the unbiased probability distribution along the CV under a specified prior;^27^ the results are provided in Supporting Information Section S5.

After removing equilibration timesteps, the trajectory length was cut from the beginning up to a selected percentage of the converged simulation run, from 4 to 100% (*T* = [4, 6.3, 10, 16, 25, 40, 60, 80, 90, 100]). The number of windows were varied from 3 to the maximum available of 25 (*W* = [3, 4, 6, 8, 10, 13, 16, 19, 22, 25]). A *T* × *W* grid was constructed for a total of 100 UQ calculations. Note that independent simulation replicas would provide a stronger test of statistical variability; however, their generation was computationally prohibitive for the systems considered here.

Five separate calculations were performed for each grid evaluation. One used an evenly spaced subset of umbrella windows, while the remaining four were randomly selected subsets of the available 25 windows. RMSE with respect to a full data set WHAM reference and predictive standard deviation were calculated for each reconstruction and averaged over these five computations. Averaging over window selection was introduced to mitigate bias associated with the window selection process, which in general may be different depending on the Bayesian optimization (or equivalent) decision-theoretic scheme employed.

Heatmaps of the average RMSE and standard deviations were constructed to visualize the relationship between error and predictive uncertainty. Log–log parity plots were used to compare the RMSE and average predictive standard deviation across all 100 ablation conditions, with their association quantified by Pearson’s correlation coefficient (*r*) using the untransformed values. A common threshold of 5 kJ/mol was used to identify accurate but underconfident (U) and inaccurate but overconfident (O) reconstructions.

Predictive accuracy and sharpness were jointly assessed using the continuous ranked probability score (CRPS), calculated pointwise relative to the WHAM reference and averaged over the free energy profile and ablation grid. For methods with Gaussian predictive marginals, the pointwise CRPS was

30 $$\begin{array}{l} \mathrm{CRPS}\left(F_{j},F_{j}^{\mathrm{ref}}\right)=s_{j}\left[z_{j}\left(2\Phi \left(z_{j}\right)-1\right)+2\phi \left(z_{j}\right)-\frac{1}{\sqrt{\pi }}\right]\\ z_{j}=\frac{F_{j}^{\mathrm{ref}}-{\mathrm{F̅}}_{j}}{s_{j}} \end{array}$$

where *F̅*_*j*_ and *s*_*j*_ are the predictive mean and standard deviation at grid point *j*, *F*_*j*_^ref^ is the WHAM reference free energy, and ϕ and Φ are the standard normal density and distribution functions. When *s*_*j*_ = 0, the score reduces to | *F̅*_*j*_ – *F*_*j*_^ref^|. A comparison between the LOO pseudolikelihood and marginal likelihood is provided in Supporting Information Section S6.

## Results

### Noise as an Uncertain Hyperparameter

GP-based free energy methods typically estimate histogram and derivative noise variances from simulation statistics and condition the GP posterior on these fixed values. However, this implicitly assumes that the prior is sufficiently expressive to fit the training data such that any remaining discrepancy between the observations and GP can be attributed to statistical uncertainty in MD sampling alone. In general, this assumption need not hold, since the posterior free energy profile is coupled to both the MD data and the GP prior. Treating the noise variance as known therefore forces the GP to explain any residual data-model mismatch through its remaining hyperparameters, collapsing the hyperposterior into unlikely regions of the hyperparameter space.

The consequences of this approximation are shown in generalized hyperposterior MAP summarized in Table 1 and hyperposterior distributions visualized in Figure 2. For a fixed noise model, the hyperposterior over the l and *w* hyperparameters is multimodal, with a MAP estimate of l=0.10nm and *w* = 1.88 kJ/mol (Figure 2a). The second mode collapses to an unrealistically short l=0.01nm accompanied by an inflated kernel amplitude *w* = 3.95 kJ/mol. The consequences of this bimodality are clear in the sampled GP posterior, with an RMSE of 9.20 kJ/mol and predictive standard deviation of 7.97 kJ/mol. Small discrepancies between the plotted posterior histograms and the reported MAP estimates arise because the MAP values were obtained by direct optimization of the joint objective, rather than by estimating marginal modes from binned histograms. The histogram shapes therefore provide a visual summary of the sampled marginal distributions, but their bin-dependent maxima should not be interpreted as the joint MAP.

**Figure 2:**
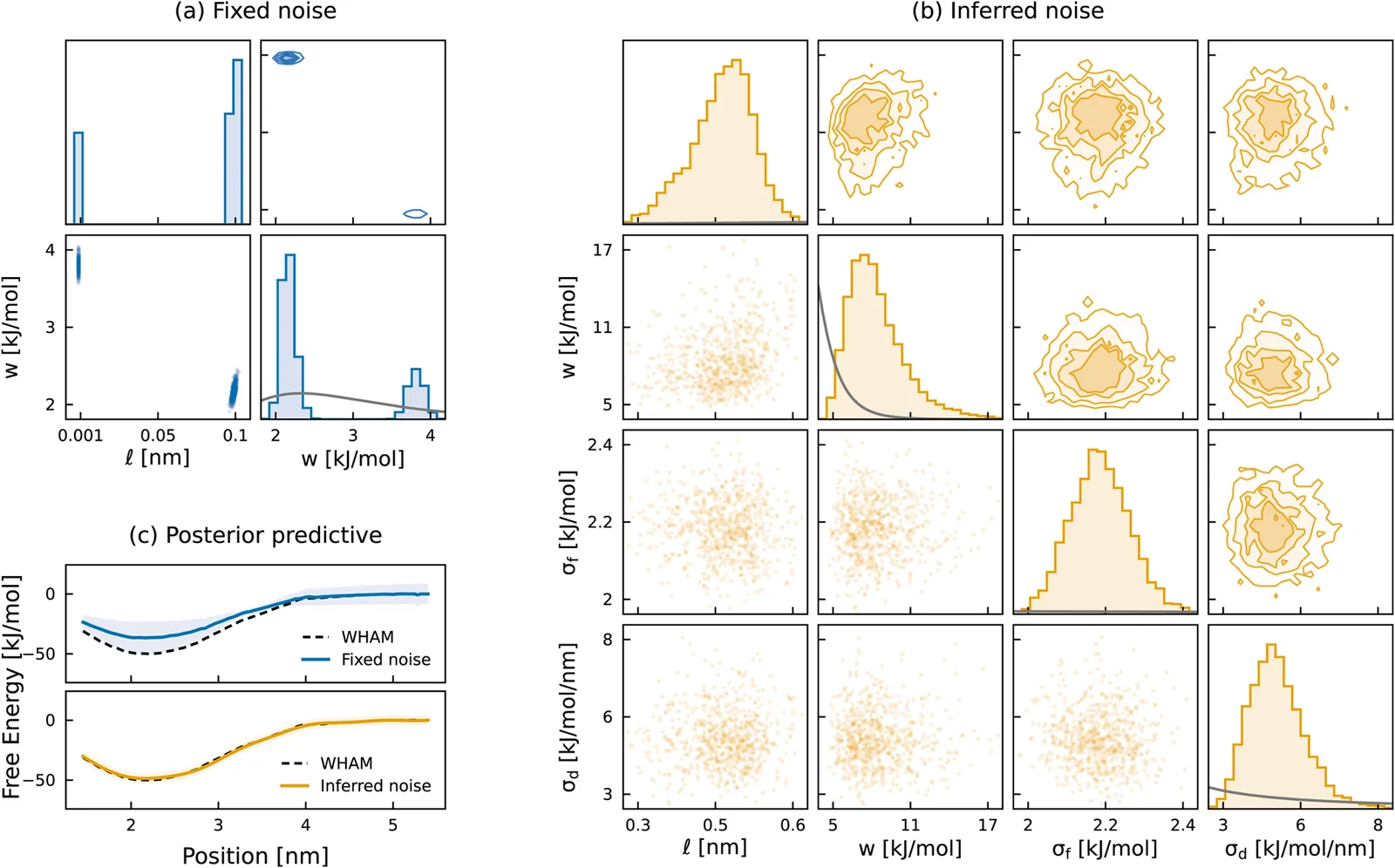
Comparison between fixed and inferred noise hyperparameter models. (a) Corner plot of fixed noise hyperposterior with marginal hyperpriors (gray line). (b) Corner plot of inferred noise hyperposterior. Raw HMC-NUTS samples below diagonal and sample contour map above diagonal. (c) Posterior predictive surfaces comparing the fixed and inferred noise models with shaded region given by ±σ.

**Table 1:** LOO MAP Hyperparameter Estimates for Fixed- and Inferred-Noise GP Models

Noise model	l	*w*	σ_f_	σ_d_
Fixed	0.10	1.88	–	–
Inferred	0.48	7.51	2.18	4.94

Relaxing the delta-function priors on the nuisance parameters σ_f_ and σ_d_ acknowledges that statistical uncertainty from the enhanced sampling MD is not the only source of discrepancy between the observations and the GP model. As shown in Figure 2b, jointly inferring these quantities removes the pathological short-length-scale mode and yields a well-behaved hyperposterior centered around l=0.48nm and *w* = 7.2 kJ/mol. The inferred values of σ_f_ and σ_d_ are larger than those predicted from simulation statistics alone, indicating that the model attributes part of the residual discrepancy to limitations of the GP representation rather than forcing it into the kernel hyperparameters. This change also resolves the deterioration in predictive RMSE and average standard deviation, with values of 1.59 and 4.12 kJ/mol, respectively. Modeling the histogram and derivative noise variances as inferred hyperparameters therefore appears to correct hyperparameter identifiability and uncertainty propagation pathologies from the generalized hyperposterior. This idea represents an important conceptual departure of our framework from prior work.

### Uncertainty Evolution with Data Acquisition

An important property of reliable UQ methods is that the estimated uncertainty should be large when the prediction is poor, and only become small when the prediction is accurate. This behavior ensures that we are close to an optimal min-max problem, where we minimize computational time while still obtaining accurate and trustworthy predictions. We evaluated four UQ methods on our R9 umbrella sampling data set to assess their ability to reproduce this desirable behavior by knocking out umbrella windows and trajectory time. Heatmaps and log–log parity plots of average RMSE and standard deviation for these data ablation grids are shown in Figure 3.

**Figure 3:**
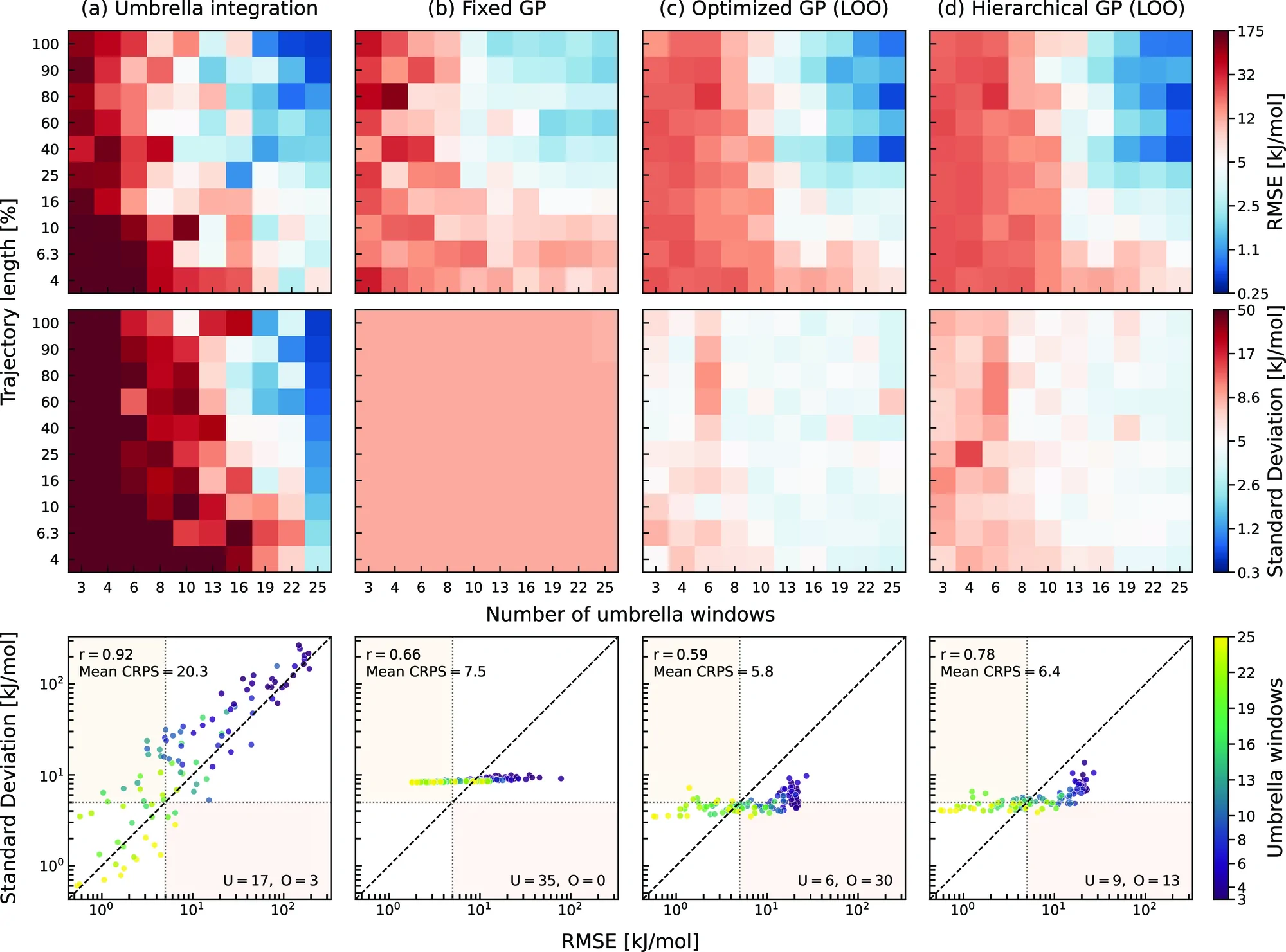
Data ablation heatmap grids. Columns represent different free energy profile reconstruction and error estimation methods: (a) Umbrella Integration (UI) with the block averaging, (b) GP with fixed hyperparameters, (c) optimized hierarchical GP model with LOO pseudolikelihood and (d) sampled hierarchical GP model with LOO pseudolikelihood. The first row shows the mean RMSE with respect to the reference WHAM prediction, while the second row shows the average predictive standard deviation in the given model. Color bars are shared within each row and shown on a logarithmic scale. White denotes a chemical accuracy threshold (RMSE = 5 kJ/mol, top row; average SD = 5 kJ/mol, second row). The third row shows parity plots between average RMSE and average SD for all 100 runs, along with a Pearson correlation coefficient, mean pointwise CRPS, and numbers of underconfident (U) and overconfident (O) predictions based on the 5 kJ/mol threshold, indicated by the yellow and red shaded regions, respectively.

All methods considered show the expected trend that increasing the amount of simulation data improves reconstruction accuracy, resulting in lower average RMSE values (top row of Figure 3). Furthermore, the RMSE of the free energy prediction was excellent in the high-data limit for all tested methods. For this particular system, increasing the number of umbrella windows provided a larger improvement than extending the trajectory length within individual windows. However, this observation is system dependent and should not be interpreted as a general rule.

The second row of Figure 3 shows heatmaps of the average predictive standard deviation for each method, where red indicates values greater than 5 kJ/mol and blue values below this threshold. For short trajectories and low window counts, UI with block averaging produces large standard deviations, reaching values up to 265 kJ/mol. Large uncertainty estimates persist even in regions of the grid corresponding to 8–22 umbrella windows, where the free energy profile mean is already within chemical accuracy. As additional data are introduced, the standard deviation estimates collapse abruptly toward near-zero values. This behavior suggests that the UI + block averaging uncertainty estimates may provide a misleading picture of convergence. Specifically, this method is susceptible to underconfidence, in that it will begin to achieve high accuracy (low RMSE) before the predictive uncertainties converge, with 17 underconfident predictions identified. The parity plot in the bottom row shows that UI + block averaging performs well in correlating predicted and realized errors with a Pearson correlation of *r* = 0.92, but tends to be either inaccurate or excessively uncertain as indicated by its large mean CRPS of 20.3 kJ/mol.

The fixed-hyperparameter GP (fixed GP) model produces a different pathology. The uncertainty remains nearly unchanged regardless of the amount of available data (8.3–9.2 kJ/mol), as evidenced by the essentially uniform standard deviation heatmap and nearly horizontal parity plot. Consequently, the predictive uncertainty becomes largely decoupled from the information content of the simulations and cannot reliably indicate whether additional sampling has improved the reconstruction. Our particular choice of hyperparameters resulted in 35 underconfident predictions and a large improvement in mean CRPS of 7.5 kJ/mol, indicating that the GP largely corrects excessive broadness of the predictive distribution observed for UI + block averaging.

In contrast, the hierarchical GP models provide uncertainty estimates that evolve consistently with the amount of available data. The optimized GP (Figure 3c) showed an improved mean CRPS of 5.8 kJ/mol, but is overconfident in the low-data setting (30 overconfident simulations), with a predictive standard deviation approximately 18% lower, on average, than the hyperposterior-propagated model (Supporting Information Section S7). Propagating hyperparameter uncertainty with the hierarchical GP corrects this overconfidence (Figure 3d), improving error-ranking (*r* = 0.78) compared to the other GP models, retaining an acceptable mean CRPS of 6.4 kJ/mol, and significantly reducing the number of overconfident predictions compared to the optimized GP from 30 to 13. This comparison indicates that hyperparameter uncertainty significantly contributes to predictive uncertainty, and the parity plots show that this effect is strongest in the high-error (or equivalently, low-data) regime. The data also show that GP-based free energy estimators tend to underestimate uncertainty even when hyperparameter uncertainty is accounted for, suggesting that a richer probabilistic formulation may be required to achieve excellent uncertainty calibration.

### Propagating Free Energy Uncertainty

Access to a probability distribution enables principled uncertainty propagation to downstream quantities-of-interest (QoIs). Consequently, uncertainties in the simulation data, model assumptions, and GP hyperparameters can be propagated directly to these observables, yielding predictive distributions rather than point estimates.

Figure 4 shows uncertainty propagation to the free energy barrier height of the R9-membrane interaction as a function of the number of umbrella windows. The barrier-height distributions reveal the same qualitative behavior observed for the free energy profiles. UI with block averaging, in line with the previous heatmap, produces broad barrier-height distributions for low window counts, reflecting substantial uncertainty in the reconstructed surface. The distribution remains broad until a nearly complete data set is available, at which point it abruptly collapses to an almost delta-function-like distribution centered on a single barrier value.

**Figure 4:**
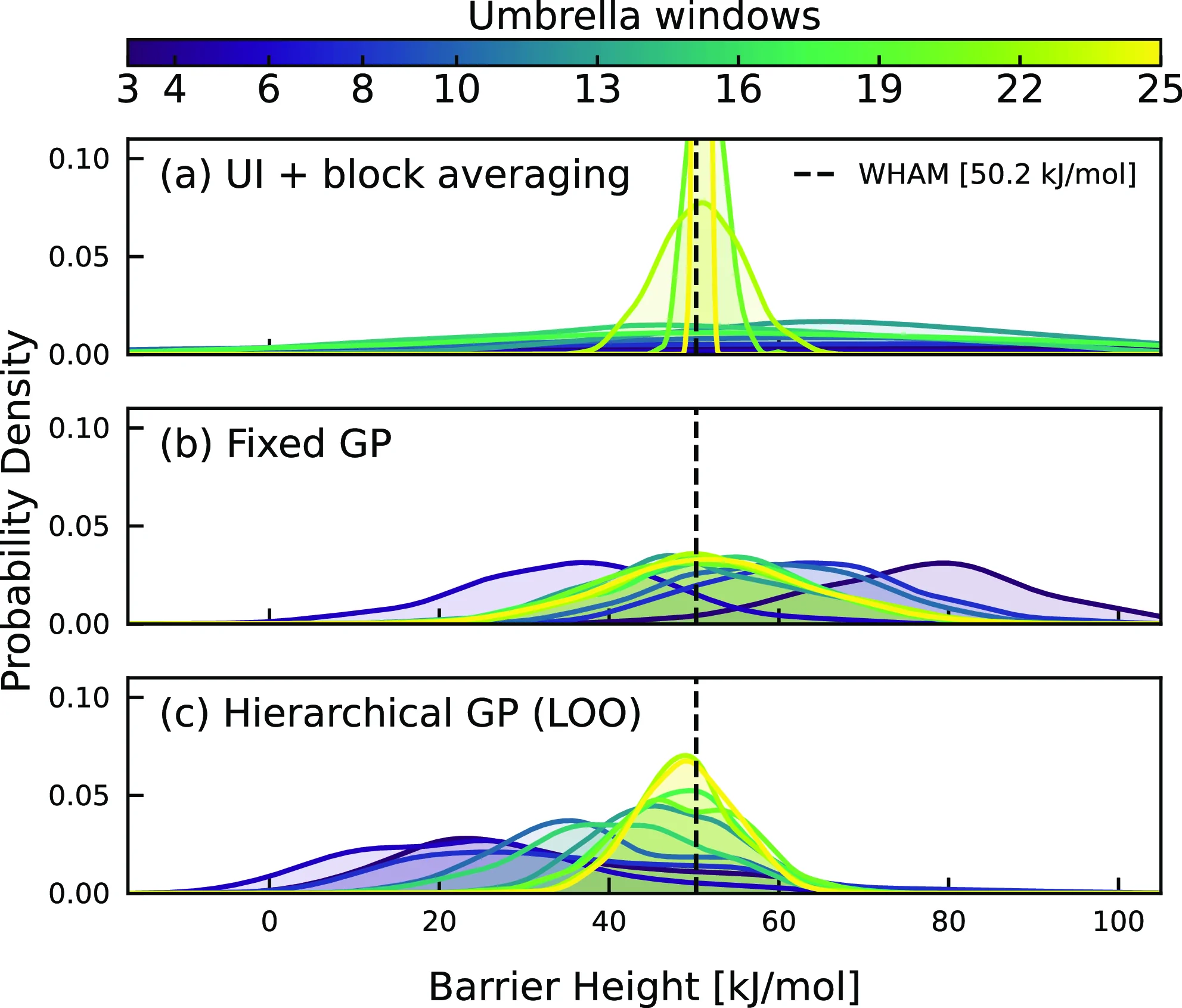
Barrier-height distributions for three PMF estimation methods as a function of sampling window count (100% trajectory length) for (a) UI with block averaging, (b) the fixed GP, and (c) the hierarchical GP. Distributions correspond to the free energy barrier height, Δ*A* ^‡^ = max (*f*) – min (*f*), estimated from 500 samples at each window count and colored from 3 (purple) to 25 (yellow) windows. The vertical dashed line indicates the WHAM reference barrier height.

For the fixed GP, the width of the barrier-height distribution remains nearly unchanged as additional data are acquired. Although the mean barrier estimate gradually approaches the reference value, the associated uncertainty does not evolve with the amount of information provided by the simulations. Consequently, the uncertainty estimate offers little indication of whether the calculation has converged.

In contrast, the hierarchical GP exhibits a smooth and physically sensible convergence behavior. The barrier-height distribution systematically narrows around the reference value while remaining appropriately broad in undersampled regimes. This indicates that the model is able to adapt its uncertainty estimates to the amount of available information, yielding both accurate barrier predictions and reasonable uncertainty estimates throughout the convergence process.

An interesting observation comparing the fixed and hierarchical GP models is the tendency for the hierarchical GP to underpredict the barrier height, particularly with sparse window sampling. We attribute this behavior to the GP prior, which recall in this case is the zero function. When the data provide weak constraints, i.e., with low window/sample counts, the posterior tends toward the mean, resulting in a reduced barrier height. Marginalizing over uncertain hyperparameters makes this prior-driven behavior more apparent in poorly sampled regimes, whereas the fixed GP can predict either higher or lower barriers depending on its training data. A small underestimation persists with the full data set, although it is minor relative to the width of the barrier-height distribution.

### Extended Lagrangian Metadynamics

Although the preceding results focused on an umbrella sampling example, the hierarchical GP formulation is applicable to data obtained from any enhanced sampling method. To illustrate this, we applied our framework to extended Lagrangian metadynamics simulations of phenol interacting with a lipid bilayer. As in the previous case, the CV is defined as the distance between the centers of mass of the membrane and the phenol molecule normal to the membrane surface. The primary distinction from umbrella sampling is that histogram and derivative observations are not naturally partitioned into independent umbrella windows, but must instead be constructed by discretizing the metadynamics trajectory. This additional preprocessing step introduces another layer of modeling assumptions, making metadynamics a useful test of the generality of the framework. Details of the molecular simulations and discretization procedure are provided in Supporting Information Section S8.

In analogy to the umbrella-sampling analysis, we examined how uncertainty changes with the retained length of the metadynamics trajectory. The results for the fixed and hierarchical GP are shown in Figure 5. The fixed GP behaved similarly to the umbrella sampling case, showing improving RMSE with additional trajectory data, decreasing from 11.30 to 1.41 kJ/mol, but nearly constant average predictive standard deviation, varying only from 8.89 to 8.33 kJ/mol. Thus, the fixed GP model did not adapt its uncertainty scale appreciably as additional metadynamics data were included.

**Figure 5:**
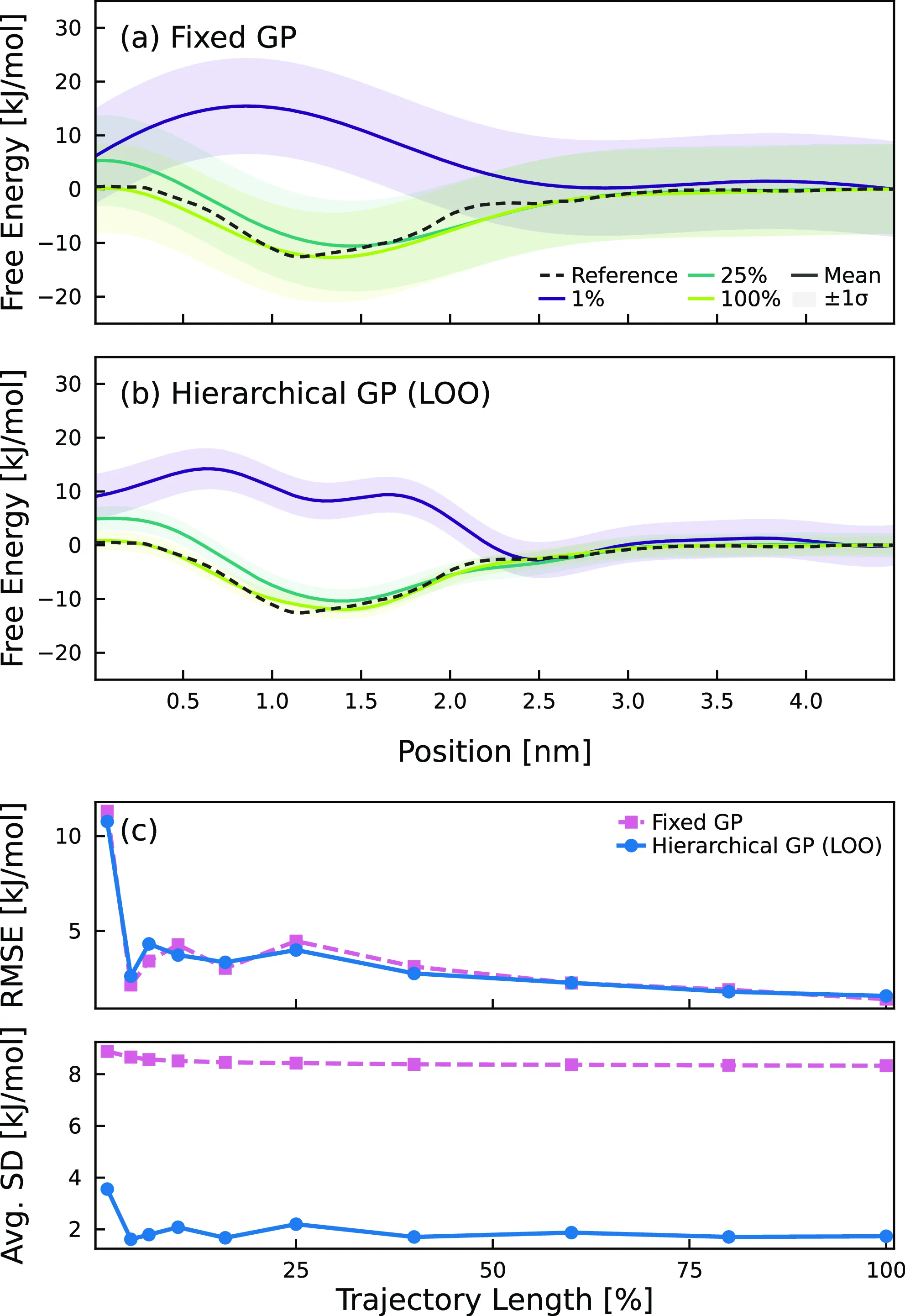
Metadynamics GP-based free energy reconstruction as a function of trajectory length. (a) Fixed GP and (b) hierarchical GP posterior predictive means are shown relative to the WHAM reference for selected trajectory fractions; shaded regions denote ±σ. (c) Shows the RMSE and average predictive standard deviation as a function of retained trajectory length for the fixed GP and hierarchical GP models.

In contrast, the hierarchical GP produced uncertainty estimates that changed with the amount of available data and remained broadly correlated with reconstruction accuracy. For the hierarchical GP, the average predictive standard deviation decreased rapidly from 3.56 kJ/mol at 1% of the trajectory to approximately 1.6–2.2 kJ/mol over the remaining trajectory lengths. Over the same range, the RMSE decreased from 10.77 kJ/mol at 1% trajectory length to 1.58 kJ/mol for the full trajectory. The average predictive standard deviation was correlated with the predictive mean error, with a Pearson correlation coefficient of ρ = 0.95.

These results suggest that, despite the additional modeling assumptions required to discretize metadynamics trajectories, the hierarchical GP framework can still provide reliable UQ beyond umbrella sampling. The fixed GP remains a useful baseline, but its uncertainty evolution is substantially less responsive.

## Discussion

The results from the previous sections reinforce a simple but important lesson: uncertainty quantification for free energy profiles is inseparable from the assumptions of the underlying statistical model. Consequently, strong assumptions about GP hyperparameters inevitably propagate into the predictive uncertainties. In this study, we showed that the treatment of noise, the choice of likelihood (or pseudolikelihood) objective, and uncertainty in the GP hyperparameters can alter predictive uncertainty estimation for free energy reconstruction, even in simple one-dimensional problems. Our results suggest that reliable free energy profiles, accompanied by meaningful uncertainty estimates, can often be reconstructed from substantially less data than is required by conventional free energy reconstruction methods. This may be particularly valuable for *ab initio* molecular dynamics (AIMD), where the computational expense of generating sufficiently converged free energy landscapes remains a major practical limitation.

In the context of automated free energy workflows, omitting sources of uncertainty becomes particularly problematic because the simulation data are sparse, noisy, and highly dependent on sampling protocol. We showed that generalized hierarchical Bayesian inference can mitigate this problem by propagating uncertainty in the GP hyperparameters directly into the predictive distribution. As a result, the model is able to adapt to the available information and provides a more reliable measure of convergence. This behavior was demonstrated for umbrella sampling and extended Lagrangian metadynamics examples, but in principle is applicable to any free energy method, including the accelerated weighted histogram,^7^ alchemical decoupling,^9^ and multistate Bennet acceptance ratio^14^ methods. These results may also inform existing GP-based free energy methods, such as BayesMBAR,^14^ that use marginal likelihood-based inference to optimize hyperparameters without propagating their uncertainty.

The hierarchical GP framework has its limitations, perhaps the most significant being computational cost. Unlike conventional GP-based free energy reconstruction, which requires only a single evaluation of the predictive posterior at fixed hyperparameters, this approach requires repeated assembly and factorization of a joint covariance matrix throughout HMC sampling of the hyperposterior. For the one-dimensional problems considered here, this additional cost is negligible compared to that of generating well-sampled MD trajectories, but could become a limiting factor for high-dimensional surfaces. We demonstrate extension to two-dimensional surfaces using precomputed umbrella sampling of alanine dipeptide backbone torsions^28^ in Supporting Information Section S9, for which hierarchical GP reproduced the central UQ trends observed for the one-dimensional systems. For the one-dimensional R9 data set, the fixed GP, optimized GP, and hierarchical GP reconstructions required approximately 4.6 s, 14.3 s, and 5.0 min of wall-clock time, respectively, corresponding to an approximately 64-fold cost increase for hierarchical inference relative to fixed-hyperparameter inference. Hardware and detailed timings across representative data ablation conditions are provided in Supporting Information Section S10. Furthermore, if one needs to increase the flexibility of the GP prior (which typically introduces additional hyperparameters), the computational burden associated with Bayesian inference will also increase. Developing scalable inference strategies for richer GP models therefore remains an important area for future work.

Beyond computational considerations, the reliability of any Bayesian inference procedure is ultimately limited by the assumptions encoded in the prior model. In the present work, we considered only stationary covariance kernels, which assume that correlations depend solely on distance in CV space and not on location. While this assumption is common, there is no physical reason why a free energy profile should exhibit uniform correlation structure throughout an entire CV domain. Regions near metastable basins, transition states, and steep free energy barriers may possess fundamentally different characteristic length scales and smoothness properties. A natural extension of the present framework would therefore be the use of nonstationary kernels, either in parametric form ref^29^ or fully nonparametric form ref.^30^ Such models would permit spatially varying correlation structures and potentially provide more realistic uncertainty estimates, albeit at the cost of a substantially more complex hyperposterior inference problem.

At the same time, a GP-based representation of free energy profiles creates opportunities for integration with a broader ecosystem of Bayesian methodologies in computational chemistry. GPs have already been successfully applied to scattering analysis,^29^ force field optimization,^23,31–33^ and machine-learning interatomic potentials.^34–36^ Representing free energy profiles within the same probabilistic framework raises the possibility of combining information from multiple experimental and computational sources into unified Bayesian models. Such approaches could enable consistency checks between scattering data, electronic structure calculations, force field models, and free energy calculations, while also providing a mechanism for propagating uncertainty between different levels of theory.

## Conclusions

We evaluated several uncertainty quantification strategies for free energy calculations and found that generalized hierarchical GP inference was the only approach whose uncertainty estimates consistently adapted to the amount and quality of the available simulation data. In contrast, existing methods exhibited systematic failure modes by conditioning on fixed modeling assumptions, including fixed hyperparameters and noise models that attribute all uncertainty to statistical sampling. These results highlight the importance of propagating uncertainty throughout the statistical model rather than conditioning on point estimates or fixed probabilistic assumptions. Although important challenges remain—including computational scaling, higher-dimensional collective variable spaces, and more expressive nonstationary covariance models—we conclude that hierarchical GP inference provides a promising foundation for next-generation automated free energy calculations in which uncertainty quantification is treated as a primary objective rather than an afterthought.

## Supplementary Material



## Data Availability

The source code used in this study, including the associated calculation outputs and artifacts, is archived on Zenodo at 10.5281/zenodo.22797928. A lightweight version for ongoing use and development is available on GitHub at https://github.com/brennonshanks/freeGP. The simulation data sets are archived at 10.5281/zenodo.21358802. A tutorial included in the GitHub repository allows users to test the code on a synthetic system, and the repository README provides guidance for preparing umbrella-sampling and metadynamics data for use with the software. Additional information is available from the corresponding authors upon reasonable request.

## Notes

Gaussian process hyperposterior specification and fixed-hyperparameter reference model; sensitivity to the length-scale hyperprior; HMC-NUTS convergence diagnostics; umbrella-integration uncertainty estimation using block averaging; comparison of hyperparameter optimization with hyperposterior sampling; comparison of marginal likelihood and leave-one-out pseudolikelihood objectives; molecular simulation details for the metadynamics calculation; and analysis of the metadynamics binning and discretization procedure (PDF).
