# Supplementary material for: Uncertainty Quantification for Free Energy Calculations by Generalized Hierarchical Bayesian Inference
Source: J Chem Theory Comput. 2026 Oct 21;22(21):11221–32. doi: 10.1021/acs.jctc.6c01533 (PMC13757756; doi:10.1021/acs.jctc.6c01533)
Supplement: Supplementary file 1 [file ct6c01533_si_001.pdf]

Uncertainty quantification for free energy calculations by generalized hierarchical Bayesian inference

Martin Skorna, Adam Gottfried, Zuzana Janackova, Katarina Baxova, Pavel Jungwirth, and Brennon L. Shanks*
Institute of Organic Chemistry and Biochemistry of the Czech Academy of Sciences, Flemingovo nám. 2, 166 10 Prague 6, Czech Republic

(*Electronic mail: shanks.brennon@uochb.cas.cz)

(Dated: 17 September 2026)

S1. HYPERPOSTERIOR SPECIFICATION

The GP was assigned a squared-exponential covariance function prior,

$$k_{\mathrm{SE}}(\boldsymbol{\xi},\boldsymbol{\xi}') = w^2 \exp\left(-\frac{|\boldsymbol{\xi}-\boldsymbol{\xi}'|^2}{2\ell^2}\right). \tag{S1}$$

where ℓ is the correlation length and w is the kernel amplitude. All positive hyperparameters were sampled in natural-log space. Expressing distances in nm and free energies in kJ/mol, the default independent hyperpriors were

$$\begin{aligned}\log\ell &\sim \mathcal{N}(\log 4,\ 1^2),\\ \log w &\sim \mathcal{N}(1,\ 0.5^2),\\ \log\sigma_{\mathrm{f}} &\sim \mathcal{N}(0.5,\ 2^2),\\ \log\sigma_{\mathrm{d}} &\sim \mathcal{N}(0.5,\ 2^2).\end{aligned}$$

Equivalently, ℓ, w, σ_{f}, and σ_{d} follow log-normal distributions with medians of 4 nm, 2.72 kJ/mol, 1.65 kJ/mol, and 1.65 kJ/mol/nm, respectively. The length-scale prior was intentionally chosen to be weakly informative, allowing the posterior to learn substantially shorter or longer correlation lengths when supported by the data. The parameters σ_{f} and σ_{d} represent nuisance noise scales whose variances enter the diagonal of the function and derivative observation covariance matrices. Their priors were intentionally broad to permit uncertainty arising from both sampling variability and model mismatch.

Unless otherwise stated, these hyperpriors were combined with the LOO pseudo-likelihood to construct the generalized hyperposterior.

A. Details of the Fixed Hyperparameter GP

The fixed-hyperparameter reference used the same stationary covariance with $\ell = \pi/2$ nm and $w = 4.184\sqrt{10}$ kJ/mol (the heuristic recommendation is $\sqrt{10}$ kcal/mol[S1]). Although these kernel parameters were fixed, the observation covariance was estimated from each umbrella trajectory. After equilibration removal, the CV time series $\mathbf{x}$ in each window was approximated as an autoregressive process of order one (AR(1)). This provides an inexpensive approximation to the integrated autocorrelation time. The lag-one coefficient was estimated using a weakly regularized ratio of the lag-one sample autocovariance to the sample variance,

$$\hat{\phi} = \tanh\left[\frac{\sum_t (x_t-\bar{x})(x_{t+1}-\bar{x})}{\lambda + \sum_t (x_t-\bar{x})^2}\right],$$

with $\lambda = 0.01$ in the squared coordinate units of the input trajectory. The regularization shrinks poorly determined estimates toward zero and the tanh transformation constrains $\hat{\phi}$ to the stationary interval $(-1,1)$. The corresponding AR(1) statistical inefficiency and effective sample size were

$$\tau = \frac{1+\hat{\phi}}{1-\hat{\phi}}, \qquad N_{\mathrm{eff}} = \frac{N}{\tau}.$$

We did not cap N_{eff} at N; therefore, windows with negative lag-one correlation can have $\tau < 1$ and $N_{\mathrm{eff}} > N$. In practice, this estimator was used only to scale the trajectory-derived observation covariances for the fixed hyperparameter GP and umbrella integration references, and should be regarded as an approximate AR(1)-based correction rather than a full integrated autocorrelation time analysis.

For a histogram bin with probability p_i, uncertainty in the histogram-derived free-energy observation was represented by

$$\mathrm{Var}(F_i) = \frac{1}{\beta^2 N_{\mathrm{eff}}}\left(\frac{1}{p_i}-1\right). \tag{S2}$$

The multinomial constraint introduces covariance between bins from the same umbrella window,

$$\mathrm{Cov}(F_i,F_j) = -\frac{1}{\beta^2 N_{\mathrm{eff}}}, \qquad i\neq j.$$

For the derivative observation obtained from an umbrella with force constant k and positional variance s_x^2, the estimated variance was

$$\mathrm{Var}(\overline{F'}) = \frac{k^2 s_x^2}{N_{\mathrm{eff}}}. \tag{S3}$$

B. Hyperprior Sensitivity Analyses

Bayesian inference is influenced by prior selection, making sensitivity analyses important for assessing robustness. Since it is computationally impractical to exhaustively explore all hyperprior choices, we focus on

a case-study of the length scale ℓ. ℓ is a particularly relevant parameter to investigate, because larger length scales regularize non-physical free energy fluctuations by favoring smoother surfaces. We therefore examined several length-scale hyperpriors in a data-limited regime (7 windows, 25% trajectory), where the influence of prior assumptions should be most apparent.

Four priors were examined: (i) a prior uniform in $\log \ell$, (ii) the default prior $\log \ell \sim \mathcal{N}(\log 4, 1^2)$ used throughout this work, and two informative priors centered at $\ell = 0.5$ nm with standard deviations of 0.5 and 0.1 in log space. Priors on all remaining hyperparameters were unchanged.

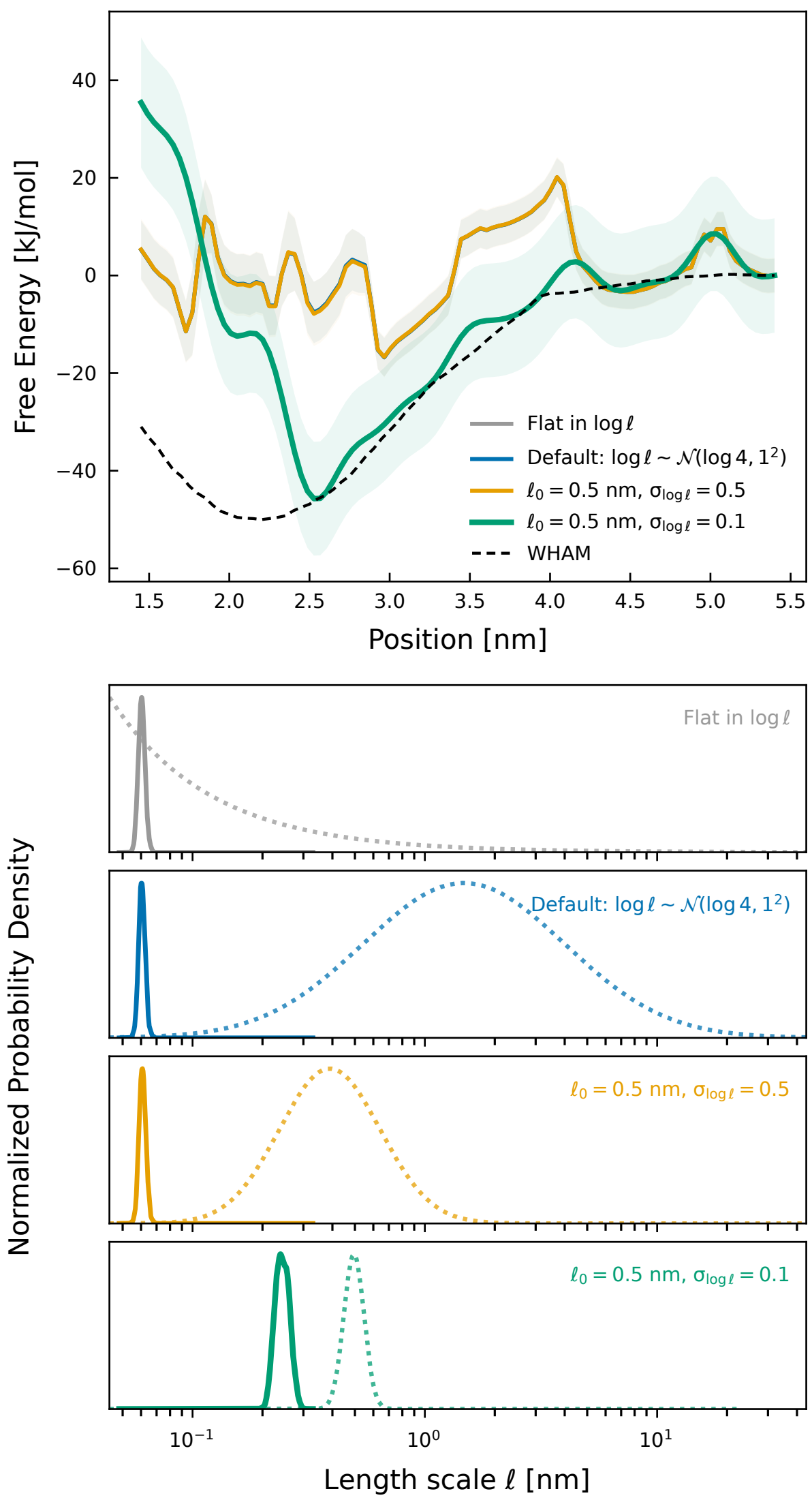

FIG. S1. Length-scale hyperprior sensitivity analysis. Top: Posterior free energy profile compared to WHAM on the full umbrella sampling dataset. Shaded regions represent the predictive credible interval of $\pm\sigma$. Bottom: Normalized ℓ priors (dashed) and sampled hyperposterior marginals (solid).

The reconstruction was largely insensitive to the uniform, default, and moderately informative priors (Table S1), suggesting a data-dominated generalized posterior. The inferred posterior median length scales differed by less than 1%, their credible intervals strongly overlapped, and the resulting free energy surfaces were visually indistinguishable. Across these three cases, the mean predictive standard deviation ranged from 4.71-4.92 kJ/mol and the RMSE relative to the UI reference ranged from 17.88-17.94 kJ/mol.

On the other hand, the strongly informative prior centered at 0.5 nm with $\sigma_{\log} = 0.1$ shifted the median ℓ to 0.244 nm. This stronger regularization suppresses short-length-scale structure, but also increased the RMSE relative to UI to 29.98 kJ/mol and increased the mean predictive standard deviation to 11.88 kJ/mol. These results suggest that the free energy reconstruction is robust to broad and moderately informative choices of the length-scale hyperprior, but strongly informative priors can significantly impact the reconstruction and thus should be used with care.

TABLE S1. Posterior sensitivity to tested ℓ hyperpriors.

Prior	Median ℓ (nm)	RMSE	SD
Uniform in $\log \ell$	0.0604	17.94	4.71
$\log \ell \sim \mathcal{N}(\log 4, 1^2)$	0.0605	17.94	4.72
$\log \ell \sim \mathcal{N}(\log 0.5, 0.5^2)$	0.0609	17.88	4.92
$\log \ell \sim \mathcal{N}(\log 0.5, 0.1^2)$	0.2443	29.98	11.88

S2. HAMILTONIAN MONTE CARLO DIAGNOSTICS

To assess convergence of our HMC-NUTS implementation, we systematically varied burn-in period and number of retained posterior samples for the R9 dataset. Four data regimes were considered: an *easy* case consisting of 25 umbrella windows and the full post-equilibration trajectory, a *medium* case with 13 windows and 50% of each trajectory, a *hard* case with 7 windows and 25% of each trajectory, and a *super-hard* case with 3 windows and 10% of each trajectory. For each regime, four independent chains were run using 250, 500, or 1000 burn-in steps and 250, 500, or 1000 retained samples per chain, yielding a total of 36 calculations. All calculations employed the squared-exponential kernel with inferrable noise hyperparameters, default hyperpriors, and the LOO pseudo-likelihood.

Convergence was assessed using the potential scale reduction factor ($\hat{R}$), effective sample size (ESS), the number of divergent transitions, and visual inspection of chain trace plots and marginal posterior distributions. In addition, posterior-derived quantities, including the average predictive standard deviation and free-energy barrier height, were monitored across run lengths to assess sensitivity of physically relevant observables to HMC-NUTS settings.

The chains were well mixed and no divergent transitions were observed. For the easy and medium regimes, all calculations yielded excellent convergence diagnostics, with maximum $\hat{R}$ values of 1.0062 and 1.0033, respectively. In the hard regime, eight of the nine runs satisfied $\hat{R} < 1.01$, with the only exception corresponding to the shortest calculation (250 burn-in steps and 250 retained samples per chain), for which the maximum $\hat{R}$ was 1.0196 and the minimum ESS was 232.

The super-hard regime provided the most stringent test because the limited amount of data weakly constrained the hyperposterior. Seven of the nine runs achieved $\hat{R} < 1.01$. The two failed cases both used only 250 retained samples, yielding maximum $\hat{R}$ values of 1.0107 and 1.0110. In contrast, all calculations using 500 or more retained samples satisfied $\hat{R} < 1.005$, while all calculations using 1000 retained samples satisfied $\hat{R} < 1.002$, irrespective of burn-in length. Thus, increasing the number of retained samples per chain is more beneficial than extending the burn-in period beyond 250 steps for weakly identified posteriors.

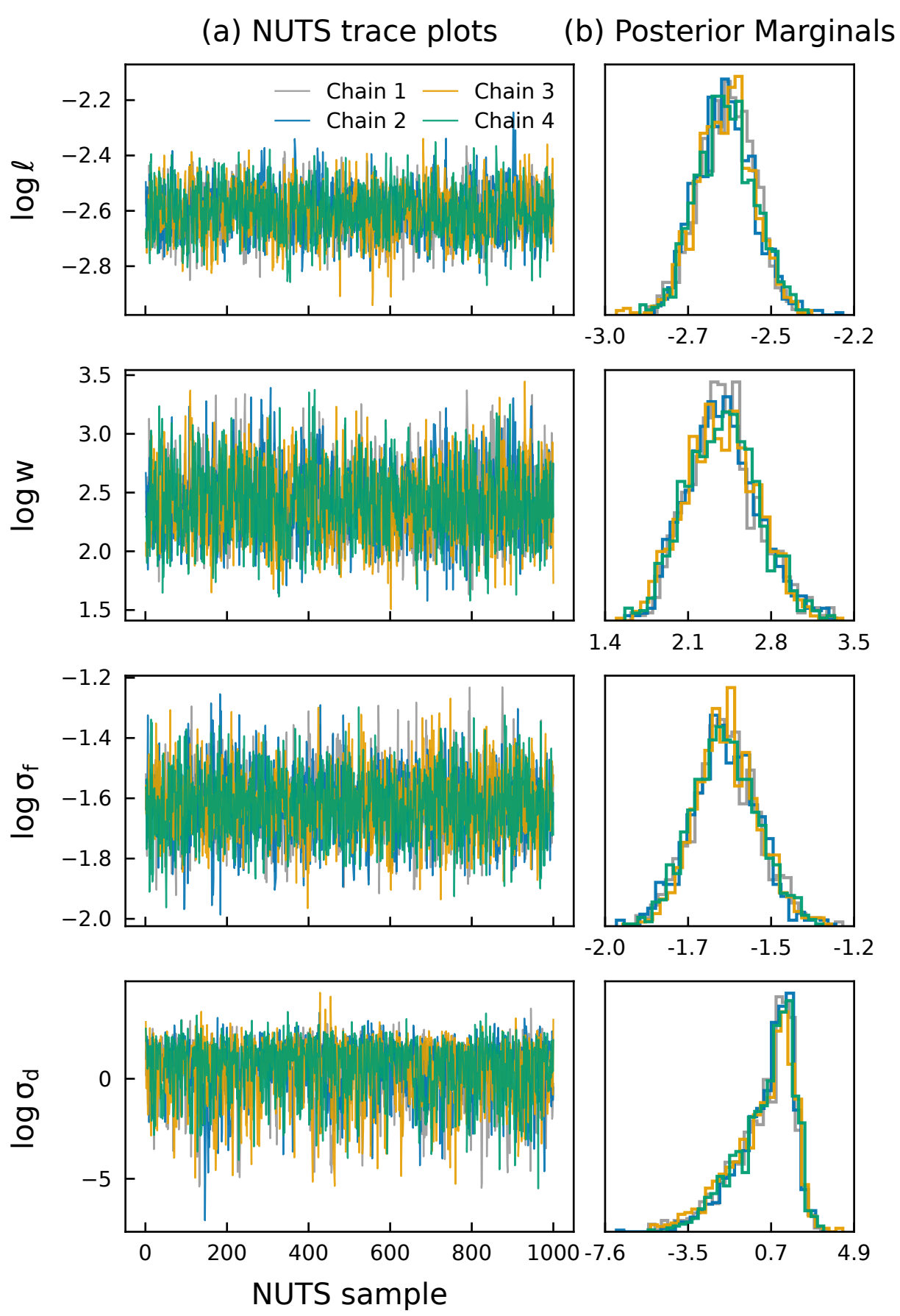

FIG. S2. NUTS chain tracking for the super-hard data case (3 windows, 10% trajectory) exhibits excellent mixing and sampling of the hyperposterior despite limited data.

Production calculations were therefore performed using 500 burn-in steps followed by 1000 retained samples per chain. At this setting, all four data regimes exhibited excellent convergence (Table S2), with a maximum $\hat{R}$ of 1.0024, a minimum ESS of 1479, and no divergent transitions. Trace plots showed stationary fluctuations without persistent chain offsets, and marginal posterior distributions exhibited strong overlap across chains (Figure S2).

TABLE S2. NUTS diagnostics and divergences (Divs) using a 500 step burn-in and 1000 retained samples for four chains.

Windows	Trajectory	Max $\hat{R}$	Min ESS	Divs
25	1.00	1.0008	2823	0
13	0.50	1.0012	2393	0
7	0.25	1.0024	1554	0
3	0.10	1.0018	1479	0

Posterior-derived observables were similarly insensitive to the precise burn-in and sampling length once standard convergence criteria were satisfied. Across all nine burn-in/sample combinations, the coefficient of variation of the mean predictive standard deviation was 1.66%, 1.31%, 1.84%, and 2.93% for the easy, medium, hard, and super-hard regimes, respectively. Corresponding free-energy barrier estimates varied only between 48.57-48.69, 50.40-50.72, 36.84-36.91, and 30.46-30.94 kJ/mol. The largest within-regime variation was therefore only 0.49 kJ/mol, observed for the super-hard case.

S3. REFERENCE SIMULATION CONVERGENCE TESTS

The convergence of the R9–lipid bilayer MD simulations was assessed by examining overlap between neighboring window histograms, convergence of the WHAM free energy profile, and the stability of the R9 radius of gyration throughout the trajectories. Following removal of the 10 ns equilibration period, the autocorrelation time of each window was estimated using the procedure described in Section S1, and the trajectories were thinned using a stride equal to the ceiling of the estimated autocorrelation time. The aggregate histogram support was continuous across the analyzed reaction coordinate (Figure S3a). We additionally reconstructed the WHAM free energy profile using the first 20, 40, 60, 80, and 100% of each trajectory. The cumulative profiles approached the full-data reference as additional trajectory data were included, with the 80% reconstruction differing from the full-data profile by an RMSE of only 0.21 kJ/mol (Figure S3b). Finally, we examined the R9 radius of gyration as a function of simulation time as a complementary measure of conformational stability and to identify possible slowly sampled degrees of freedom orthogonal to the biased coordinate. The radius of gyration was stable across the trajectories, with the exception of a slow relaxation apparent in the first window (Figure S3c), where R9 is

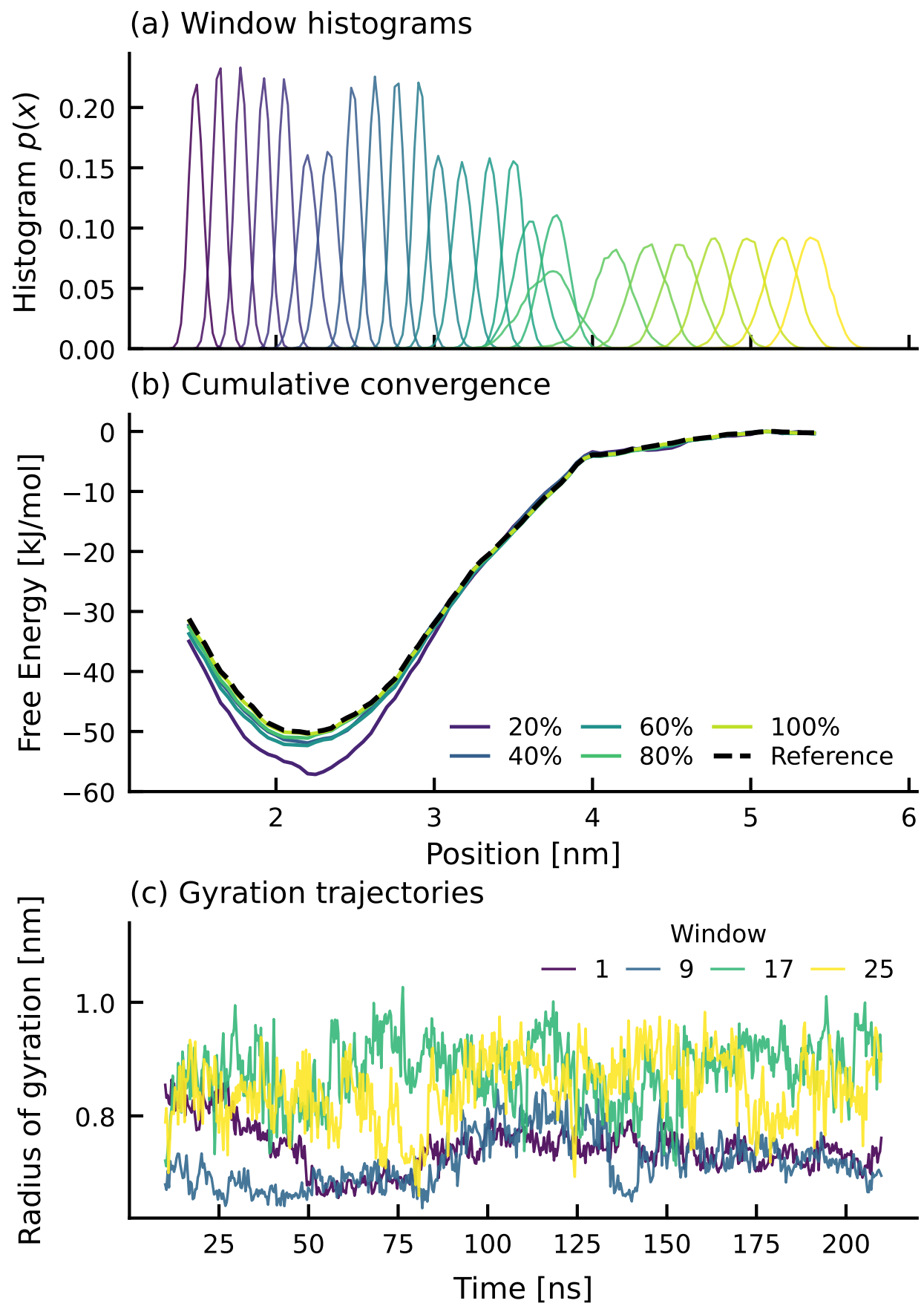

FIG. S3. Convergence assessment of the WHAM reference surface for the R9 system. (a) Autocorrelation-thinned coordinate histograms for the 25 umbrella windows. Each histogram is normalized independently and vertically offset according to its umbrella center. (b) WHAM surfaces reconstructed from progressively longer trajectories. (c) Time series of radius of gyration for four windows representing different stages of the simulation (1: R9 embedded in membrane, 9: R9 near membrane surface, 17: near R9 detachment from membrane, and 25: R9 far from membrane).

embedded within the membrane. The histogram overlap, convergence of the reconstructed free energy profile, and stability of the peptide conformation indicate that the full-data profile provides a sufficiently converged reference for evaluating the UQ methods while retaining realistic sampling challenges present in biomolecular simulations.

S4. UMBRELLA INTEGRATION WITH BLOCK AVERAGING

After equilibration removal and trajectory truncation, each umbrella trajectory was divided into 10 contiguous blocks. For each umbrella window i and block b, a Gaussian approximation to the biased coordinate distribution was used to estimate the block-specific mean-force contribution, $A'_{i,b}(x)$. The window-specific mean-force estimate was then obtained by averaging over the block estimates,

$$\overline{A'_i}(x) = \frac{1}{B}\sum_{b=1}^{B} A'_{i,b}(x), \tag{S4}$$

where $B = 10$ is the number of blocks. The uncertainty in the window mean force was estimated from the squared standard error (SE),

$$\mathrm{SE}^2[A'_i(x)] = \frac{\mathrm{Var}_b\left[A'_{i,b}(x)\right]}{B}. \tag{S5}$$

The window-specific mean forces were combined using the standard UI weights,

$$\overline{A'}(x) = \sum_i \tilde{w}_i(x)\, \overline{A'_i}(x), \tag{S6}$$

where $\tilde{w}_i(x)$ are the normalized UI weights. Assuming independent uncertainties between umbrella windows, the variance of the combined mean force was approximated as

$$\mathrm{Var}\left[\overline{A'}(x)\right] \approx \sum_i \tilde{w}_i(x)^2\, \mathrm{SE}^2[A'_i(x)]\,. \tag{S7}$$

The PMF was obtained by trapezoidal quadrature of the mean-force profile,

$$A(x_j) = A(x_0) + \sum_{k=1}^{j} \frac{x_k - x_{k-1}}{2}\left[\overline{A'}(x_{k-1}) + \overline{A'}(x_k)\right]. \tag{S8}$$

The pointwise mean-force variance was propagated through the same trapezoidal integration procedure using a running sum to estimate the within-replicate free energy variance. This propagation assumes that mean-force uncertainties at different grid points are independent and therefore neglects covariance between neighboring mean-force estimates. For each data condition, multiple independent window selections were analyzed. The total UI uncertainty was computed as the sum of the average within-replicate variance and the variance across replicate window selections, consistent with the law of total variance.

S5. DATA ABLATION WITH BAYESWHAM

To compare our results on GP-based estimators with other Bayesian inference implementations, we evaluated BayesWHAM[S2] using the same combinations of trajectory length and number of umbrella windows for the R9/bilayer system, together with the same evenly spaced and randomly selected window subsets. Histogram counts were corrected for temporal correlation

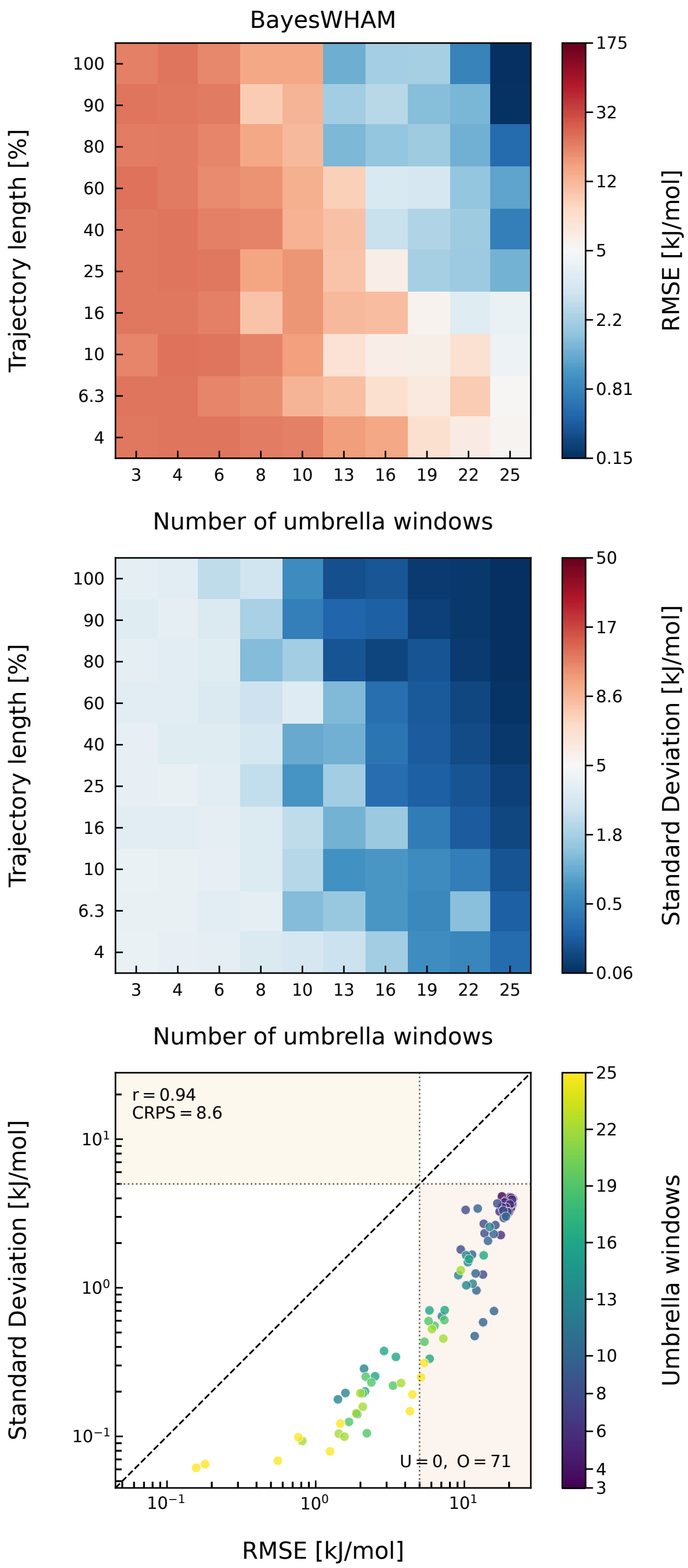

FIG. S4. BayesWHAM results for the R9 data ablation study. The upper and middle panels show the RMSE relative to the full-data WHAM reference and the mean pointwise posterior standard deviation, respectively, as functions of trajectory length and number of umbrella windows. The lower panel compares the RMSE and mean posterior standard deviation for all 100 grid points on logarithmic axes. Points are colored by the number of umbrella windows, and the dashed line indicates equal RMSE and posterior standard deviation. The dotted lines mark 5 kJ/mol; U and O denote the numbers of grid points classified as underconfident (U) and overconfident (O) using this threshold, respectively. The reported Pearson correlation coefficient and mean pointwise CRPS were calculated across the complete $T \times W$ grid.

by dividing the counts from each window by the corresponding AR(1) autocorrelation time. BayesWHAM calculations used the window-specific umbrella centers and force constants from the original simulation input files and a uniform Dirichlet prior over the unbiased histogram probabilities.

The BayesWHAM posterior was sampled using NUTS. Initial calculations used two chains with 300 warmup steps and 500 retained samples per chain. Calculations that did not satisfy the sampling diagnostics outlined in the main text were repeated using four independently initialized chains and the cell-specific WHAM MAP solution as the initial reference point. A calculation was retained when the maximum $\hat{R}$ was no greater than 1.05, the minimum effective sample size was at least 100, and no divergent transitions were observed. We relaxed the more stringent convergence criteria used in the main text ($\hat{R} < 1.01$, ESS > 200) because of the substantially higher computational cost of sampling the BayesWHAM probability distributions and the numerical instabilities encountered in many chains. These criteria retained 283 of the 460 individual calculations and provided at least one valid calculation for every point on the $T \times W$ grid. Reported values for each grid point were averaged over the window selections satisfying the sampling criteria.

Figure S4 shows the resulting error, predictive uncertainty, and their relationship across the ablation grid. The mean posterior standard deviation generally increased with reconstruction error, giving a Pearson correlation coefficient of $r = 0.94$. However, the posterior intervals were substantially narrower than the deviations from the WHAM reference. 71 of the 100 grid points had an RMSE greater than 5 kJ/mol while retaining an average posterior standard deviation below 5 kJ/mol.

For sample-based BayesWHAM posteriors, CRPS was computed empirically from posterior free-energy samples $\{F_j^{(m)}\}_{m=1}^{M}$:

$$\mathrm{CRPS}_j = \frac{1}{M}\sum_{m=1}^{M}\left|F_j^{(m)} - F_j^{\mathrm{ref}}\right| - \frac{1}{2M^2}\sum_{m=1}^{M}\sum_{m'=1}^{M}\left|F_j^{(m)} - F_j^{(m')}\right|.$$

Here, F_j^{ref} is the WHAM reference free energy at grid point j. The mean pointwise CRPS across the grid was 8.6 kJ/mol. Thus, although BayesWHAM estimated uncertainties closely correlated with observed errors, its posterior intervals were strongly overconfident.

S6. SENSITIVITY TO LIKELIHOOD OBJECTIVE

Hyperparameter inference requires defining an objective function for constructing the generalized posterior over the GP hyperparameters. The standard Bayesian

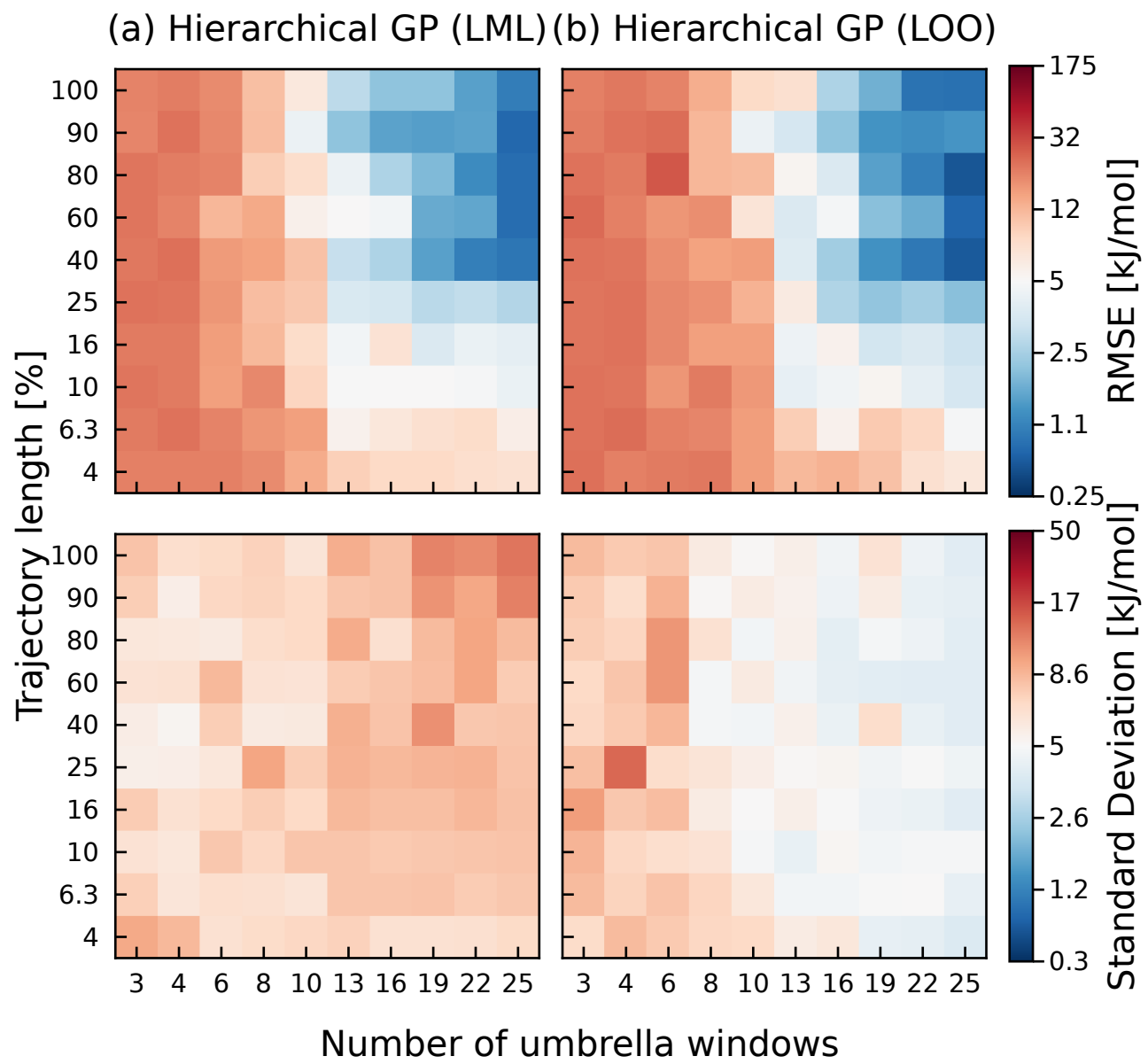

FIG. S5. Mean RMSE and SD ablation grid heat maps comparing marginal likelihood and LOO pseudo-likelihood generalized hyperposterior constructions.

choice is the marginal likelihood, which evaluates how probable the observed data are under a given set of hyperparameters after integrating over the latent free energy profile. However, maximizing the probability of the observed data is not necessarily equivalent to maximizing predictive performance on unseen data. Since the primary objective of the present work is reliable uncertainty quantification rather than reconstruction of the mean free energy surface alone, we also considered a leave-one-out (LOO) pseudo-likelihood objective.[S3] Generalized hyperposteriors constructed from both objectives were compared using identical GP priors, hyperpriors, and HMC-NUTS sampling procedures.

A comparison between the marginal likelihood and LOO hyperparameter objectives at different data conditions is shown in Figure S5. The reconstructions obtained from the two objectives are similar for the posterior mean. The primary distinction between the two approaches emerged in their uncertainty estimates. Hyperposteriors constructed using the LOO pseudo-likelihood produced uncertainty estimates that more consistently tracked the observed accuracy of the reconstructed free energy surfaces across the data ablation study. On the other hand, the marginal likelihood objective actually exhibits an increase in predictive uncertainty as more data are obtained.

This behavior is expected from the underlying objectives. The marginal likelihood rewards hyperparameters that provide a globally self-consistent explanation of the observed data under the GP model. In contrast, the LOO pseudo-likelihood directly evaluates predictive performance by repeatedly assessing how well the model predicts observations omitted during training. Because UQ is fundamentally a predictive task, the LOO objective naturally places greater emphasis on predictive reliability than on explaining the training observations themselves.

These results suggest that the choice of hyperposterior objective function can influence the practical usefulness of uncertainty estimates even when its effect on the reconstructed mean is small. Consequently, for predictive UQ we recommend the LOO pseudo-likelihood as the default objective for hyperposterior inference in GP-based free energy reconstruction.

S7. RISKS OF HYPERPOSTERIOR OPTIMIZATION

In many GP applications, hyperparameters are optimized using marginal likelihood or a predictive objective (such as LOO), with uninformative (flat) hyperpriors. Optimization locates the *maximum a posteriori* (MAP),

$$\hat{\boldsymbol{\theta}}_{\mathrm{MAP}} = \arg\max_{\boldsymbol{\theta}} p(\boldsymbol{\theta} \mid \boldsymbol{y}), \tag{S9}$$

or, in the case of the LOO objective,

$$\hat{\boldsymbol{\theta}}_{\mathrm{LOO}} = \arg\max_{\boldsymbol{\theta}} p_{\mathrm{LOO}}(\boldsymbol{y} \mid \boldsymbol{\theta}) p(\boldsymbol{\theta}). \tag{S10}$$

Here we compare GP-based free energy reconstructions obtained using optimized hyperparameters to HMC-NUTS hyperposterior sampling, see Figure S6. The mean inferred by the two approaches was similar. However, notable differences emerged in the uncertainty estimates. Hyperposterior sampling consistently produced broader predictive distributions, particularly in regions of sparse sampling where multiple hyperparameter configurations remained compatible with the available data.

From a practical perspective, the choice between optimization and hyperposterior sampling therefore depends on the intended application. When the primary goal is reconstruction of the mean, optimization is a reasonable and computationally efficient approximation. However, UQ is non-negligibly impacted by neglecting uncertainties in the underlying hyperparameters, particularly in low-data regimes.

We note that, for the systems considered in this work, the additional computational cost associated with HMC-NUTS remained negligible compared to the cost of generating MD trajectories. The principal challenge is therefore not the computational expense of the Bayesian inference itself, but rather the scaling of hyperparameter-marginalized GP models to increasingly flexible kernel representations and higher-dimensional CV spaces.

S8. METADYNAMICS

A. Molecular Simulation Details

A bilayer patch containing 128 1,2-dioleoyl-phosphatidylcholine (DOPC) molecules was generated

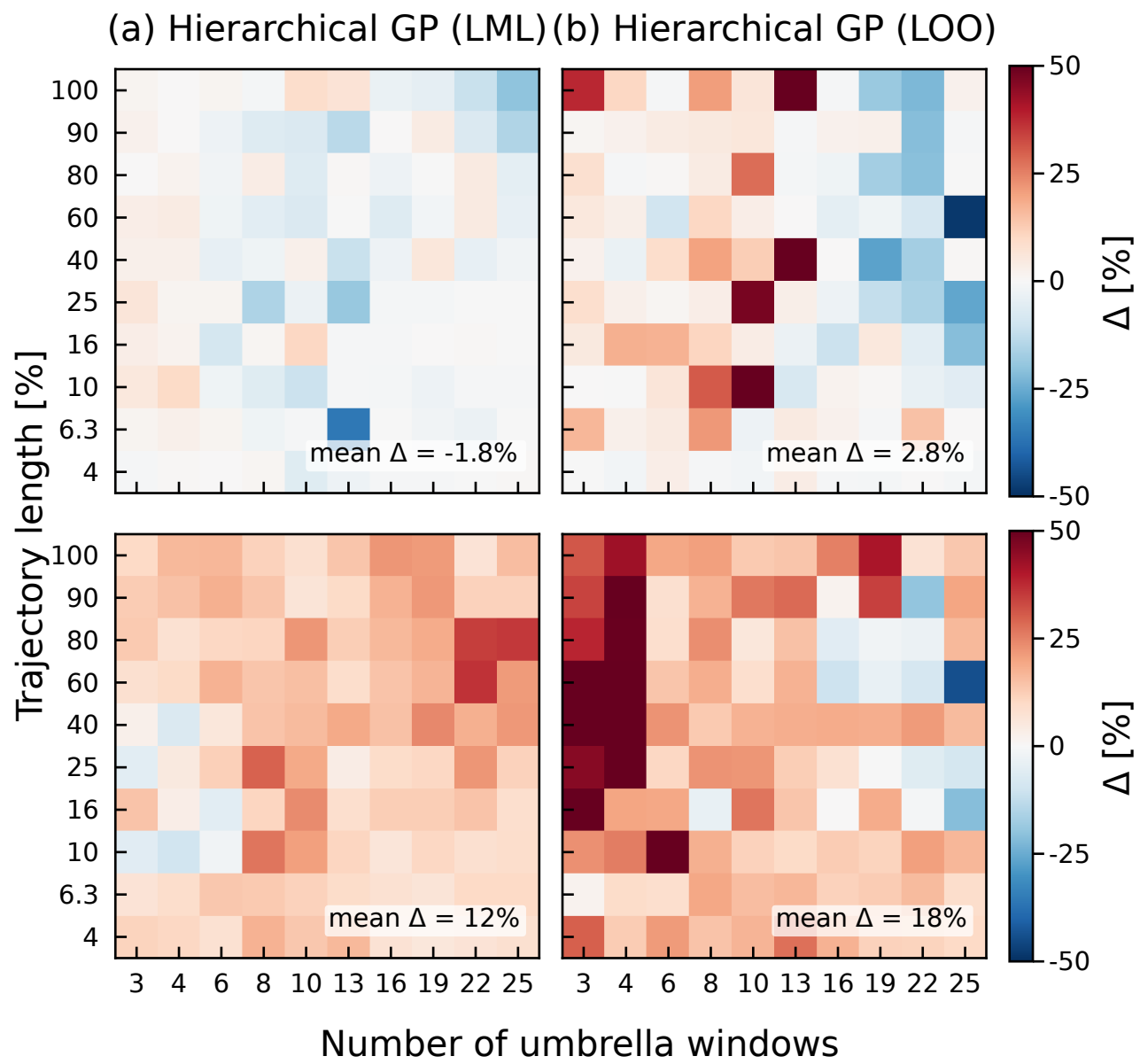

FIG. S6. Percentage difference (Δ) in mean RMSE and standard deviation between propagated hyperposteriors and MAP estimation. Blue indicates a decrease in the plotted quantity and red indicates an increase.

using CHARMM-GUI Membrane Builder.[S4–S6] The system was solvated and the ions were added to neutralize the system and mimic 0.15M NaCl solution.

All simulations were performed using GROMACS 2026.1.[S7–S9] The membrane was equilibrated using CHARMM36[S10] force field and the GROMACS input parameters recommended by CHARMM-GUI.[S11,S12] To the equilibrated system, one phenol molecule was added and the system was equilibrated for another 20 ns. The parameters of the phenol molecule were obtained using CHARMM Small Molecule Library.[S13]

The extended Lagrangian metadynamics simulation was performed using a PLUMED 2.9.3 plugin.[S14,S15] As a CV, we employed the distance between the center of mass (COM) of the membrane and the COM of the phenol molecule ranging from -1 to 5 nm. The phenol was restrained using a flat-bottom potential with the force constant of 1000 kJ/mol/nm^2. The temperature was set to 303.15 K. The coupling constant of the extended Lagrangian was 10.000 kJ/mol/nm^2 and the relaxation time was 1 ps. The Gaussian potentials were applied with a rate of 0.004 kJ/mol per picosecond and their widths were set to 0.1 nm. The length of the metadynamics simulation was 1 µs.

In all simulations, the stochastic velocity rescaling thermostat[S16] was used with a reference temperature of 303.15 K. The pressure of 1 atm was maintained by the stochastic cell rescaling barostat.[S17] The Verlet cutoff scheme was used.[S18] Force-switch cutoff of the van der Waals interactions was used between 1.0 and 1.2 nm. Long-range electrostatics was computed with the particle mesh Ewald method[S19] with the cutoff of explicit short-range electrostatic interactions at 1.2 nm. An integration step of 2 fs was used in all simulations, with the bonds to hydrogen atoms converted to rigid constraints using the LINCS algorithm[S20] and water molecules constrained using SETTLE.[S21] Periodic boundary conditions were applied in all directions.

B. Metadynamics Binning Procedure

Unlike umbrella sampling, metadynamics trajectories are not naturally partitioned into independent sampling windows and therefore require *post hoc* discretization of the history-dependent bias into histogram and derivative observations. The choice of discretization is not unique and can influence both the GP reconstruction and the inferred hyperparameter distributions. If the discretization is too fine, each observation contains too few data points, leading to unreliable estimates of the histogram densities and mean forces. This manifests as inflated inferred noise hyperparameters, particularly σ_d. Conversely, if the discretization is too coarse, many data points are merged into a single observation, reducing the spatial resolution of the observations and producing unnecessarily smooth reconstructions with increased predictive uncertainty. The objective is therefore to employ the finest discretization that remains statistically well supported by the available data.

The histogram and derivative discretizations are controlled by the number of histogram bins, n_h, and derivative windows, n_d, respectively. For the histogram observations, increasing n_h improves spatial resolution because the stiff coupling between the auxiliary coordinate z and the physical collective variable ξ confines the sampled ξ values to a narrow region within each bin. However, increasing n_h also enlarges the GP covariance matrix, eventually making GP inference computationally prohibitive. For this study, values above approximately $n_\mathrm{h} = 200$ became impractical computationally, while values below $n_\mathrm{h} \approx 100$ noticeably degraded the predictive mean and uncertainty. Within the interval $100 \leq n_\mathrm{h} \leq 200$, the reconstruction was largely insensitive to the precise choice of n_h.

The derivative discretization is instead limited primarily by the available simulation data. Choosing too few windows ($n_\mathrm{d} \lesssim 30$) smooths the mean-force observations and degrades the reconstruction, whereas choosing too many windows produces noisy estimates of the average coupling force because each window contains too few samples. This behavior is reflected by increasing values of the inferred derivative noise hyperparameter, σ_d, during hyperparameter optimization. To avoid overconfident or unstable reconstructions, we selected discretizations for which the inferred histogram and derivative noise hyperparameters remained of comparable magnitude over the full range of trajectory lengths considered.

Based on these considerations, the interval of interest

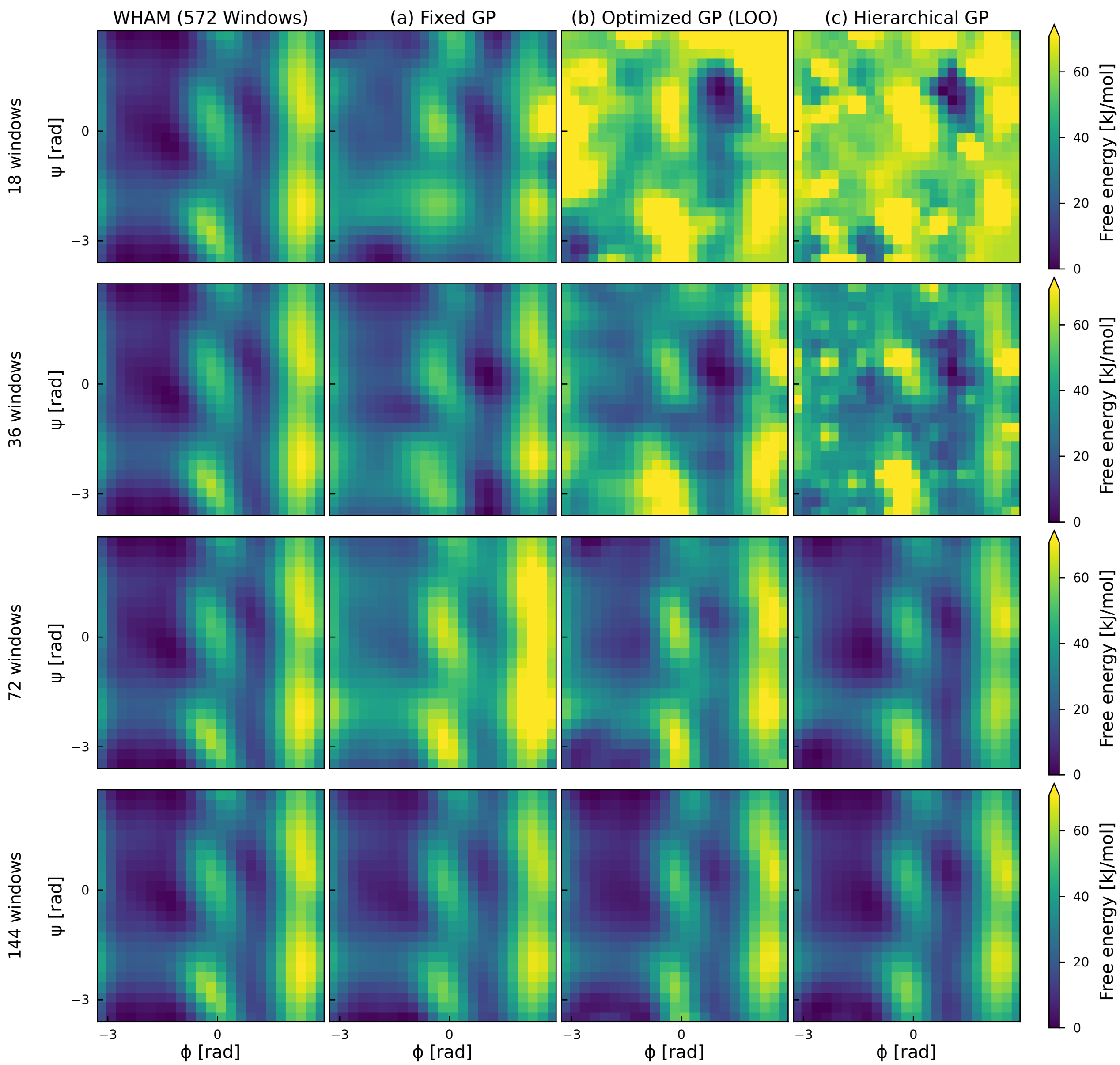

FIG. S7. Free energy heatmaps of alanine dipeptide torsion angles as a function of window count comparing the WHAM reference (with 572 windows) for the full dataset, (a) fixed GP, (b) optimized GP and (c) hierarchical GP models.

(0–4.5 nm) was discretized into $n_{\mathrm{h}} = 120$ histogram bins and $n_{\mathrm{d}} = 40$ derivative windows. Each bin and window contained approximately the same number of simulation samples, differing only in width. This equal-occupancy discretization was chosen to promote approximately homoskedastic observation noise by maintaining similar statistical precision across observations.

S9. 2D EXAMPLE: ALANINE DIPEPTIDE TORSIONS

To demonstrate that our hierarchical GP achieves similarly good UQ performance in multi-dimensional problems, we assessed two-dimensional umbrella sampling data in the backbone dihedral coordinates ϕ and ψ of alanine dipeptide.[S22] Separate reconstruction calculations were performed using reduced subsets of the full umbrella grid, corresponding to 18, 36, 72, and 144 restrained windows in two-dimensional space. Due to the high computational cost of these calculations, we did not vary trajectory length as in our one-dimensional examples. These

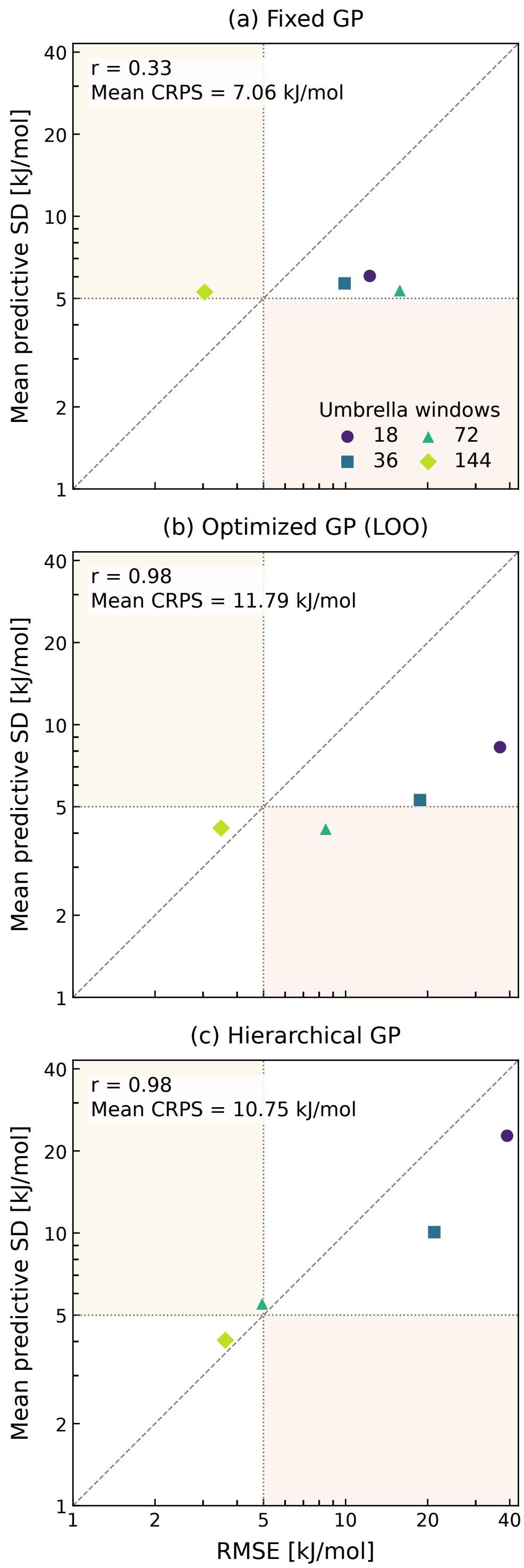

FIG. S8. Log-log parity plots of mean RMSE with respect to the converged WHAM reference versus mean predictive standard deviation for the tested cases.

subsets were compared against the full two-dimensional WHAM reference surface constructed from the complete umbrella sampling dataset.

For each selected umbrella dataset, trajectory samples were processed window by window. Because both collective variables are periodic dihedral angles, samples were wrapped using the minimum-image convention about each restraint center before constructing local histograms. Each window was converted into local histogram-based free energy observations and mean restoring force observations. These observations were then used as inputs to the GP models.

For each window subset, we performed three reconstructions: the fixed GP, MAP optimized GP, and our hierarchical GP. For the fixed GP, the length scale hyperparameter was chosen as $\ell = \pi/3$ (as used by Stecher[S1]) and $w = 20$ kJ/mol. The HMC-NUTS calculations for the GP hyperposterior were run with 300 warmup steps, 300 retained samples, four chains, and a maximum tree depth of four. Note that these settings were less conservative than the one-dimensional examples presented in the main text due to the high computational cost of the GP calculations (for 144 windows the HMC-NUTS run for 100% trajectory length completed in ∼15 hours).

The resulting free energy profiles were evaluated on a regular two-dimensional grid spanning the sampled support. Errors were computed relative to the full WHAM reference surface after applying periodic interpolation of the reference surface across the $\phi, \psi = \pm\pi$ boundaries and shifting surfaces to a common minimum. Uncertainty estimates were obtained in the same manner as the one-dimensional case, *i.e.* as the average standard deviation of the GP posterior predictive.

It is clear from Figure S7 that the GP-based methods can accurately reconstruct the two-dimensional free energy surface once sufficient data are available. At low window counts (18 and 36 windows), both the MAP and hierarchical GP models produce poor reconstructions accompanied by correspondingly large uncertainty estimates, as summarized in Figure S8, correctly indicating that additional sampling is required before the surface can be reconstructed with confidence. In contrast, the fixed GP is already relatively confident in its predictions despite an average RMSE of approximately 10 kJ/mol for both window counts.

At 72 and 144 windows, the reconstructed surfaces begin to closely approach the WHAM reference, while the MAP and hierarchical GP models exhibit correspondingly smaller uncertainty estimates. The log-log parity plots in Figure S8 further demonstrate this distinction. The fixed GP exhibits poor discrimination between low- and high-error predictions ($r = 0.33$), whereas both the MAP and hierarchical GP models show a substantially stronger relationship between predicted uncertainty and realized error ($r = 0.98$) and lie closer to parity. For this limited dataset, the mean pointwise CRPS favors the fixed GP. Of course, given the small number of ablation conditions evaluated here, these Pearson correlation coefficients and mean pointwise CRPS scores should be interpreted cautiously. These results demonstrate that the hierarchical approach extends to a multidimensional free energy surface and provides appropriately larger uncertainty when the available data are insufficient for reliable

reconstruction. While a comprehensive assessment of UQ in higher-dimensional systems would require substantially more extensive ablation studies, these results reproduce the central trends observed for the one-dimensional systems in the main text.

S10. COMPUTATIONAL COST ANALYSIS

The computational costs of UI with block averaging, fixed GP, optimized GP, and hierarchical GP inference were evaluated using three representative conditions from the R9 membrane system. These conditions correspond to the previously examined HMC calibration cases: a sparse data condition containing 3 evenly spaced umbrella windows and 10% of each trajectory, an intermediate condition containing 7 windows and 25% of each trajectory, and the full-data condition containing all 25 windows and 100% of each trajectory.

All methods were evaluated using the same input data, preprocessing procedure, and grid of 100 prediction points. MAP inference used 250 optimization steps and three independent restarts. Hierarchical inference used four HMC chains, each with 500 warmup steps and 1,000 retained samples, followed by 100 hyperposterior predictive samples. The four HMC chains were executed as separate, parallel processes. Calculations were performed on an Apple MacBook Pro with an Apple M3 Pro processor (11 CPU cores: 5 performance and 6 efficiency cores) and 18 GB of unified memory.

Wall-clock time included Python interpreter startup, trajectory loading, preprocessing, inference, prediction, and result serialization, but excluded generation of the underlying molecular dynamics trajectories. Three timed calculations were performed for each method and condition. Table S3 reports the median wall-clock time and the maximum absolute deviation from the median over three calculations. Relative costs were calculated by dividing each median time by that of the fixed GP under the same data condition.

The cost of UI and the fixed GP was equivalent across the three conditions, with the data-loading and preprocessing operations comprising a substantial fraction of their short total runtimes. MAP inference increased from approximately 6.4 s for the sparse condition to 14.3 s for the full data condition. Hierarchical inference showed the strongest dependence on problem size, increasing from approximately 28 s for the sparse condition to 298 s, or approximately 5 min, for the full condition. The hierarchical calculation was therefore approximately 5.9×, 11.0×, and 64.4× slower than the fixed GP for the sparse, intermediate, and full conditions, respectively. Although these absolute timings are specific to the hardware and implementation used here, they demonstrate the expected additional cost of repeatedly evaluating and factorizing the GP covariance structure throughout HMC sampling.

REFERENCES

[S1] T. Stecher, N. Bernstein, and G. Csányi, "Free Energy Surface Reconstruction from Umbrella Samples Using Gaussian Process Regression," J. Chem. Theory Comput. **10**, 4079–4097 (2014).

[S2] A. L. Ferguson, "BayesWHAM: A Bayesian approach for free energy estimation, reweighting, and uncertainty quantification in the weighted histogram analysis method," J. Comput. Chem. **38**, 1583–1605 (2017).

[S3] S. Sundararajan and S. S. Keerthi, "Predictive approaches for choosing hyperparameters in gaussian processes," Neural Comput. **13**, 1103–1118 (2001).

[S4] S. Jo, T. Kim, V. G. Iyer, and W. Im, "CHARMM-GUI: A web-based graphical user interface for CHARMM," J. Comput. Chem. **29**, 1859–1865 (2008).

[S5] E. L. Wu, X. Cheng, S. Jo, H. Rui, K. C. Song, E. M. Dávila-Contreras, Y. Qi, J. Lee, V. Monje-Galvan, R. M. Venable, J. B. Klauda, and W. Im, "CHARMM-GUI Membrane Builder toward realistic biological membrane simulations," J. Comput. Chem. **35**, 1997–2004 (2014).

[S6] S. Jo, J. B. Lim, J. B. Klauda, and W. Im, "CHARMM-GUI Membrane Builder for Mixed Bilayers and Its Application to Yeast Membranes," Biophys. J. **97**, 50–58 (2009).

[S7] D. Van Der Spoel, E. Lindahl, B. Hess, G. Groenhof, A. E. Mark, and H. J. C. Berendsen, "GROMACS: Fast, flexible, and free," J. Comput. Chem. **26**, 1701–1718 (2005).

[S8] M. J. Abraham, T. Murtola, R. Schulz, S. Páll, J. C. Smith, B. Hess, and E. Lindahl, "GROMACS: High performance molecular simulations through multi-level parallelism from laptops to supercomputers," SoftwareX **1-2**, 19–25 (2015).

[S9] S. Páll, M. J. Abraham, C. Kutzner, B. Hess, and E. Lindahl, *Solving Software Challenges for Exascale* (Springer International Publishing, 2015) pp. 3–27.

[S10] J. Huang and A. D. MacKerell Jr, "CHARMM36 all-atom additive protein force field: Validation based on comparison to NMR data," J. Comput. Chem. , 2135–2145 (2013).

[S11] B. R. Brooks, C. L. Brooks III, A. D. Mackerell Jr., L. Nilsson, R. J. Petrella, B. Roux, Y. Won, G. Archontis, C. Bartels, S. Boresch, A. Caflisch, L. Caves, Q. Cui, A. R. Dinner, M. Feig, S. Fischer, J. Gao, M. Hodoscek, W. Im, K. Kuczera, T. Lazaridis, J. Ma, V. Ovchinnikov, E. Paci, R. W. Pastor, C. B. Post, J. Z. Pu, M. Schaefer, B. Tidor, R. M. Venable, H. L. Woodcock, X. Wu, W. Yang, D. M. York, and M. Karplus, "CHARMM: The biomolecular simulation program," J. Comput. Chem. **30**, 1545–1614 (2009).

[S12] J. Lee, X. Cheng, J. M. Swails, M. S. Yeom, P. K. Eastman, J. A. Lemkul, S. Wei, J. Buckner, J. C. Jeong, Y. Qi, S. Jo, V. S. Pande, D. A. Case, C. L. I. Brooks, A. D. J. MacKerell, J. B. Klauda, and W. Im, "CHARMM-GUI Input Generator for NAMD, GROMACS, AMBER, OpenMM, and CHARMM/OpenMM Simulations Using the CHARMM36 Additive Force Field," J. Chem. Theory Comput. **12**, 405–413 (2016).

[S13] S. Kim, J. Lee, S. Jo, C. L. Brooks, H. S. Lee, and W. Im, "CHARMM-GUI ligand reader and modeler for CHARMM force field generation of small molecules: CHARMM-GUI Ligand Reader and Modeler for CHARMM Force Field Generation of Small Molecules," J. Comput. Chem. **38**, 1879–1886 (2017).

[S14] M. e. a. Bonomi, "Promoting transparency and reproducibility in enhanced molecular simulations," Nat. Methods **16**, 670–673 (2019).

[S15] G. A. Tribello, M. Bonomi, D. Branduardi, C. Camilloni, and G. Bussi, "PLUMED 2: New feathers for an old bird," Comput. Phys. Commun. **185**, 604–613 (2014).

[S16] G. Bussi, D. Donadio, and M. Parrinello, "Canonical sampling through velocity rescaling," J. Chem. Phys. **126**, 014101 (2007).

[S17] M. Bernetti and G. Bussi, "Pressure control using stochastic cell rescaling," The J. Chem. Phys. **153**, 114107 (2020).

[S18] L. Verlet, "Computer "Experiments" on Classical Fluids. I.

TABLE S3. Wall-clock cost of free energy reconstruction for R9 dataset conditions. Times are reported as the median ± maximum absolute deviation from the median over three calculations. Values in parentheses give the mean runtime relative to the fixed-hyperparameter GP for the same condition.

Data condition	UI	Fixed GP	Optimized GP	Hierarchical GP
Sparse	4.70 ± 0.04 s (0.98×)	4.82 ± 0.16 s (1.00×)	6.34 ± 0.13 s (1.32×)	28.77 ± 1.64 s (5.97×)
Intermediate	4.41 ± 0.01 s (0.98×)	4.52 ± 0.01 s (1.00×)	6.28 ± 0.07 s (1.39×)	50.64 ± 2.28 s (11.22×)
Full	4.63 ± 0.18 s (1.00×)	4.63 ± 0.02 s (1.00×)	14.31 ± 0.22 s (3.09×)	298.15 ± 0.67 s (64.46×)

Thermodynamical Properties of Lennard-Jones Molecules," Phys. Rev. **159**, 98–103 (1967).
[S19] T. Darden, D. York, and L. Pedersen, "Particle mesh Ewald: An N log(N) method for Ewald sums in large systems," J. Chem. Phys. **98**, 10089–10092 (1993).
[S20] B. Hess, H. Bekker, H. J. C. Berendsen, and J. G. E. M. Fraaije, "LINCS: A linear constraint solver for molecular simulations," J. Comput. Chem. **18**, 1463–1472 (1997).
[S21] S. Miyamoto and P. A. Kollman, "Settle: An analytical version of the SHAKE and RATTLE algorithm for rigid water models," J. Comput. Chem. **13**, 952–962 (1992).
[S22] B. W. Zhang, S. Arasteh, and R. M. Levy, "The UWHAM and SWHAM software package," Sci. Rep. **9**, 2803 (2019).
